# Structural analysis of fungal CENP-H/I/K homologs reveals a conserved assembly mechanism underlying proper chromosome alignment

**DOI:** 10.1093/nar/gky1108

**Published:** 2018-11-08

**Authors:** Liqiao Hu, Hao Huang, Mohan Hei, Yang Yang, Sheng Li, Yunshan Liu, Zhen Dou, Mengying Wu, Jie Li, Guang-zhong Wang, Xuebiao Yao, Hong Liu, Xiaojing He, Wei Tian

**Affiliations:** 1 Key Laboratory of Molecular Biophysics of the Ministry of Education, College of Life Science and Technology, Huazhong University of Science and Technology, Wuhan 430074, China; 2 Department of Pharmacology, University of Texas Southwestern Medical Center, Dallas, TX 75390, USA; 3 Shanghai Institute for Advanced Immunochemical Studies, ShanghaiTech University, Shanghai 201210, China; 4 Anhui Key Laboratory for Cellular Dynamics & Chemical Biology, Hefei National Research Center for Physical Sciences at the Microscale, University of Science & Technology of China, Hefei 230027, China; 5 CAS Key Laboratory of Computational Biology, CAS-MPG Partner Institute for Computational Biology, Shanghai Institute of Nutrition and Health, Shanghai Institutes for Biological Sciences, University of Chinese Academy of Sciences, Chinese Academy of Sciences, Shanghai 200031, China; 6 Department of Biochemistry and Molecular Biology, Tulane University Health Science Center, New Orleans, LA 70112, USA; 7 The Institute for Brain Research, Collaborative Innovation Center for Brain Science, Huazhong University of Science and Technology, Wuhan 430030, China

## Abstract

The kinetochore is a proteinaceous complex that is essential for proper chromosome segregation. As a core member of the inner kinetochore, defects of each subunit in the CENP-H/I/K complex cause dysfunction of kinetochore that leads to chromosome mis-segregation and cell death. However, how the CENP-H/I/K complex assembles and promotes kinetochore function are poorly understood. We here determined the crystal structures of CENP-I N-terminus alone from *Chaetomium thermophilum* and its complex with CENP-H/K from *Thielavia terrestris*, and verified the identified interactions. The structures and biochemical analyses show that CENP-H and CENP-K form a heterodimer through both N- and C-terminal interactions. CENP-I integrates into the CENP-H/K complex by binding to the C-terminus of CENP-H, leading to formation of the ternary complex in which CENP-H is sandwiched between CENP-K and CENP-I. Our sequence comparisons and mutational analyses showed that this architecture of the CENP–H/I/K complex is conserved in human. Mutating the binding interfaces of CENP-H for either CENP-K or CENP-I significantly reduced their localizations at centromeres and induced massive chromosome alignment defects during mitosis, suggesting that the identified interactions are critical for CENP-H/I/K complex assembly at the centromere and kinetochore function. Altogether, our findings unveil the evolutionarily conserved assembly mechanism of the CENP-H/I/K complex that is critical for proper chromosome alignment.

## INTRODUCTION

To ensure faithful segregation of chromosomes, the kinetochore, a multi-subunits protein complex, must be properly assembled at centromere during cell division (1–4). The kinetochore comprised of tens of proteins is organized into several layers. The outer layer of the kinetochore is assembled by three sub-complexes known as KMN (the KNL1, MIS12 and NDC80 complexes), which provides the microtubule-binding platform for chromosomes (5–11). At the inner layer, a number of centromere proteins form a network termed CCAN (the constitutive centromere-associated network) that binds to the centromere throughout the cell cycle (12–16). In vertebrates, the CCAN consists of 16 centromere proteins that are grouped into several sub-complexes: CENP-C, CENP-L/N, CENP-H/I/K/M, CENP-T/W/S/X and CENP-O/P/Q/R/U (12,16). Orthologs of some of these sub-complexes have been identified in other species, including fungi (10,11,17–19). As a substitute for canonical H3 in nucleosomes, the histone H3 variant CENP-A deposits at centromeres nucleosomes (20–23) and initiates the CCAN assembly by binding to CENP-L/N and CENP-C (24–29). Extensive studies have established the essential role of CCAN in directing the assembly of the outer kinetochore (2,4,8,14,15). CENP-C and CENP-T act as structural platform for outer kinetochore assembly by directly interacting with the MIS12 and NDC80 complexes (27,30–38).

Many components in the CCAN are held together by a complicated protein–protein interactions network (14,15,39–46). But how these interactions assemble the CCAN complex remains incompletely understood. As the core subunits of the CCAN, CENP-H, CENP-I and CENP-K (also known as Mcm16/Ctf3/Mcm22 in *Saccharomyces cerevisiae* and Fta3/Mis6/Sim4 in *Schizosaccharomyces pombe*) assemble into a ternary complex and are essential for the integrity of the kinetochore. Loss of any of these proteins significantly compromises chromosome congression (12,16). Their centromeric localizations have also been shown to be dependent on each other (12,13,16,32,43,47,48). Another CCAN subunit CENP-M has been shown by *in vitro* reconstitution to form a stable complex with CENP-H/I/K through its interaction with the C-terminus of CENP-I. This interaction is important for the CENP-I/M localization into centromere and chromosome alignment (39,43). Although the overall organization of the CENP-H/I/K/M complex has been shown by the electron microscopy analyses at low-resolution (39), the precise molecular basis for the assembly of the CENP-H/I/K complex remains largely uncharacterized. In this study, we determined the crystal structures of CENP-I N-terminus alone from *Chaetomium thermophilum* and its complex with C-termini of CENP-H/K proteins from *Thielavia terrestris*. Based on the structural and biochemical data, we identified the interacting residues that are important for the assembly of the human CENP-H/I/K complex and centromeric localization. Our findings here establish an evolutionarily conserved assembly mechanism of the CENP-H/I/K complex essential for proper chromosome alignment and segregation.

## MATERIALS AND METHODS

### Protein expression and purification

The full-length *T. terrestris* CENP-K (*th*CENP-K) and *C. thermophilum* CENP-I residues 1–229 (*ct*CENP-I^NT^) were subcloned into the BamHI and XhoI sites of pGEX-6p-1(GE Healthcare) vector with an N-terminal GST tag. The full-length *T. terrestris* CENP-H (*th*CENP-H) was subcloned into the BamHI and XhoI sites of modified pET-28a vector (Novagen) with an N-terminal His6-SUMO tag. All *th*CENP-H and *hs*CENP-H NT mutants were gene rated with standard two-step PCR-based methods and confirmed by DNA sequencing. *th*CENP-H and *th*CENP-K were co-expressed in *Escherichia coli* BL21 (DE3) cells, cultured in Terrific Broth medium at 37°C and induced by 0.2 mM Isopropyl β-D-1-thiogalactopyranoside (IPTG) at 16°C overnight when OD600 ∼1.5 was reached. Cells were harvested and disrupted by high-pressure homogenizer (ATX Engineering) in the phosphate-buffered saline (PBS) buffer, and then clarified by centrifuged at 35 000 *g* for 45 min at 4°C. The supernatant was added GST agarose beads (GE Healthcare) and rotated at 8°C for 1 h, PBS washed the collected beads in the gravity column and tagged-free using sumo-protease overnight at 4°C. The *ct*CENP-I^NT^ was expressed and affinity purified with same method. The eluted thCENP-H/K complex mixed *ct*CENP-INT on ice for 1 h to form hybrid CENP-H/I/K complex in buffer containing 20 mM Tris pH 8.0, 50 mM NaCl, 1 mM Dithiothreitol (DTT) and then further purified by anion exchange chromatography (HiTrap Q FF, GE Healthcare) and gel filtration chromatography (superdex 200 10/300 GL, GE Healthcare). The purified fungal CENP-H/I/K complex was concentrated to 15–20 mg/ml in buffer containing 20 mM Tris pH 8.0, 150 mM NaCl, 1 mM DTT for crystallization.

For *ct*CENP-I^NT^, the eluted untagged protein from gravity column in buffer containing 20 mM Hepes pH 7.0, 50 mM NaCl,1 mM DTT further purified by cation exchange chromatography (HiTrap SP FF, GE Healthcare) and gel filtration chromatography (superdex 200 10/300 GL, GE Healthcare). The purified *ct*CENP-I^NT^ was concentrated to 5–8 mg/ml in buffer containing 20 mM Tris pH 7.6, 150 mM NaCl, 1 mM DTT for crystallization.

### Crystallization, data collection and structure determination

For Crystallization trial, 2.2 mg/ml purified *ct*CENP-I^NT^ protein crystallized at 18°C using the hanging drop vapor-diffusion method by mixing 1 µl protein solution and 1 µl reservoir solution containing 0.1 M MES pH 6.5, 40% MPD, 5% PEG8000 for ∼2 days. The purified CENP-H/I/K protein complex was concentrated to 10 mg/ml crystallized with same method in reservoir solution containing 0.1M HEPES pH 7.2 and 20% PEG8000 for ∼10 days. The crystals were cryo-protected with reservoir solution supplemented with 20% glycerol and then flash-cooled in liquid nitrogen.

Crystals of *ct*CENP-I^NT^ and CENP-H/I/K diffraction data were collected at beamline BL17U and BL18 at Shanghai Synchrotron Radiation Facility (SSRF) and processed, integrated and scaled together with HKL3000 software (49). Initial phases for seleno-methaione labeled *ct*CENP-I^NT^ were obtained by SAD in the program AutoSol of Phenix software (50). Initial phases for CENP-H/I/K protein complex were obtained by molecular replacement in the program Phaser using the structure of *ct*CENP-I^NT^ as a search model. Iterative model building and refinement were performed in the programs Coot and Phenix (50,51), respectively. Data collection and refinement statistics are summarized in Supplementary Table S1.

### Analytical ultracentrifugation (AUC)

Sedimentation velocity (SV) experiments were performed using ProteomeLab XL-I analytical ultracentrifuge (Beckman Coulter, Palo Alto, CA, USA). Protein was prepared in PBS Buffer containing 10 mM Na2HPO4, 1.8 mM KH2PO4, 2.7 mM KCl and 137 mM NaCl by size exclusion chromatography (SEC) (Superdex 200 10/300 GL, GE Healthcare) and then concentrated to 1 absorbance at 280 nm. Experiments were done at 4°C using double-sector centerpieces and sapphirine windows at 42 000 rpm for about 10 h (An-60 Ti Rotor). The volume of sample loaded in the sample cell of the centerpiece was 380 μl, while the reference cell contained 400 μl PBS Buffer. Sedimentation profiles were recorded with absorbance and/or interference optics, and these data were analyzed by SEDFIT software (52) and plotted with program GUSSI.

### *In vitro* protein binding assays

5ug GST fusion protein (GST-*ct*CENP-I^NT^, GST-*th*CENP-H, GST-*th*CENP-K full-length or truncations, and GST-*hs*CENP-K full-length or truncations) in buffer containing 20 mM Tris pH 8.0, 100 mM NaCl and 1 mM DTT was bound to equilibrated GST beads previously at 8°C for 1 h, then incubated with CENP-H or other target proteins at 8°C for 45 min, and washed three times with buffer containing 20 mM Tris pH 8.0, 500 mM NaCl, 1 mM DTT and 0.02% Triton. The proteins retained on the beads were analyzed by sodium dodecyl sulphate-polyacrylamide gel electrophoresis (SDS-PAGE). GST-tag bound beads were used as controls. The relative binding activity was measured by the amount of bound CENP-H or other target proteins normalized to that of GST-tagged protein on the GST beads.

### Mammalian cell culture and transfection

HeLa Tet-On (Invitrogen) cells were grown in Dulbecco’s modified Eagle’s medium (DMEM; Invitrogen) supplemented with 10% fetal bovine serum and 10 mM L-glutamine. To arrest cells at G1/S, cells were incubated in the growth medium containing 2 mM thymidine (Sigma) for 17 h. G2 cells were collected at 7 h after the release from thymidine arrest. Mitotic cells were obtained by adding 5 mM MG132 (Sigma) at 8 h after the release from thymidine arrest and incubating for another 2 h. Plasmid transfection was performed when cells reached a confluency of about 40–60% using the Effectene reagent (Qiagen) per manufacturer’s protocols. For RNAi experiments, the siRNA oligonucleotides were purchased from Thermo Scientific. HeLa cells were transfected using Lipofectamine RNAiMax (Invitrogen) and analyzed at 24–48 h after transfection. The sequences of the siRNAs used in this study are: siCENP-H: AGAUUGAUUUGGACAGUAU, siCENP-I: GAAGGUGUGUGACAUAUAU from Thermo Scientific (48).

### Antibodies, immunoblotting and immunoprecipitation

The commercial antibodies used in this study were: mouse anti–CENP-H (ab88593, Abcam), mouse anti–CENP-I (ab168778, Abcam), rabbit anti-CENP-K (sc-81831, Santa Cruz), rabbit anti-GFP ((in house made polyclonal antibody), mouse anti-myc (11667203001, Roche)), mouse anti–β-tubulin (T4026, Sigma-Aldrich), human CREST autoimmune sera (HCT-0100, ImmunoVision), mouse anti-α-tubulin (T9026; Sigma-Aldrich). For immunoblotting, antibodies were used at 1 μg/ml for purified and monoclonal antibodies or at 1: 1000 dilutions for crude sera.

For immunoprecipitation, anti-MYC or anti-GFP antibodies were coupled to Affi-Prep Protein A beads (Bio-Rad) at a concentration of 1 mg/ml. HeLa cells were lysed with the Lysis Buffer (25 mM Tris–HCl pH 7.5, 75 mM NaCl, 5 mM MgCl2, 0.1% NP-40, 1 mM DTT, 0.5 μM okadaic acid, 5 mM NaF, 0.3 mM Na3VO4 10 mM β-glycerophosphate and 50 units/ml Turbo-nuclease). After 2 h incubation on ice and then 10-min incubation at 37°C, the lysate was cleared by centrifugation for 20 min at 4°C at 14 000 rpm. The supernatant was incubated with the antibody beads for 2 h at 4°C. The beads were washed four times with the lysis buffer. The proteins bound to the beads were dissolved in SDS sample buffer, separated by SDS-PAGE and blotted with the appropriate antibodies.

### Immunofluorescence

For whole cell staining, cells were grown and transfected on a Lab-Tek II chamber slide. After the medium was removed, cells were pre-extracted with the PHEM buffer (60 mM PIPES, 25 mM HEPES, pH 6.9, 10 mM EGTA, 2mM MgCl2) containing 0.2% Triton X-100 for 2 min, and fixed by 4% paraformaldehyde in PBS (PBSP) for 4 min, and blocked for 30 min with PBS containing 3% BSA (PBSB). Cells were then incubated with indicated primary antibodies in PBS for 1 h at room temperature or overnight at 4°C. Cells were washed three times with PBS containing 0.1% Triton X-100 (PBST) and incubated with fluorescent secondary antibodies in PBSB for 1 h at room temperature. Slides were washed with PBS-T again and mounted in ProLong Gold Antifade reagent with DAPI (Invitrogen), mounted with Aqua-Poly/Mount (Polysciences, Inc.) and sealed with nail polish. Cells were visualized with a DeltaVision microscope system (Applied Precision). Alexa Fluor 488, 568 or 647, or DAPI florescence was observed with the appropriate filter sets. All images were acquired on the same system with a 100X NA1.4 UPLS APO objective (Olympus) in a Z-stack series of 0.2-μm intervals. All images in each experiment were taken with the same light intensity and exposure time. Images were deconvolved and projected by the Sum Intensity method in SoftWoRx (Applied Precision), and further processed and analyzed with ImageJ.

Quantification of the relative intensity of kinetochore signals was done with ImageJ. For quantification, kinetochore regions were selected based on CREST signals. The non-kinetochore region was defined as the chromatin region with the kinetochore region subtracted. A circle that enclosed CREST signals from a pair of kinetochores was drawn and set as the region of interest (ROI). The integrated density of the gray value for the selected ROI was measured from each channel. The value of object intensity was then divided by the corresponding value of CREST intensity. In total, 20 ROIs per cell chosen at random were measured. The graphs and statistics were generated with Prism (GraphPad). In all plots, each dot represents one cell. All *P*-values were calculated using the Student’s *t*-test with the Prism software (GraphPad). For presentation, images were further processed with Photoshop (Adobe).

## RESULTS

### Reconstitution and three-dimensional structures of the ternary complex of fungal CENP-H/I/K homologs

**Figure 1.**
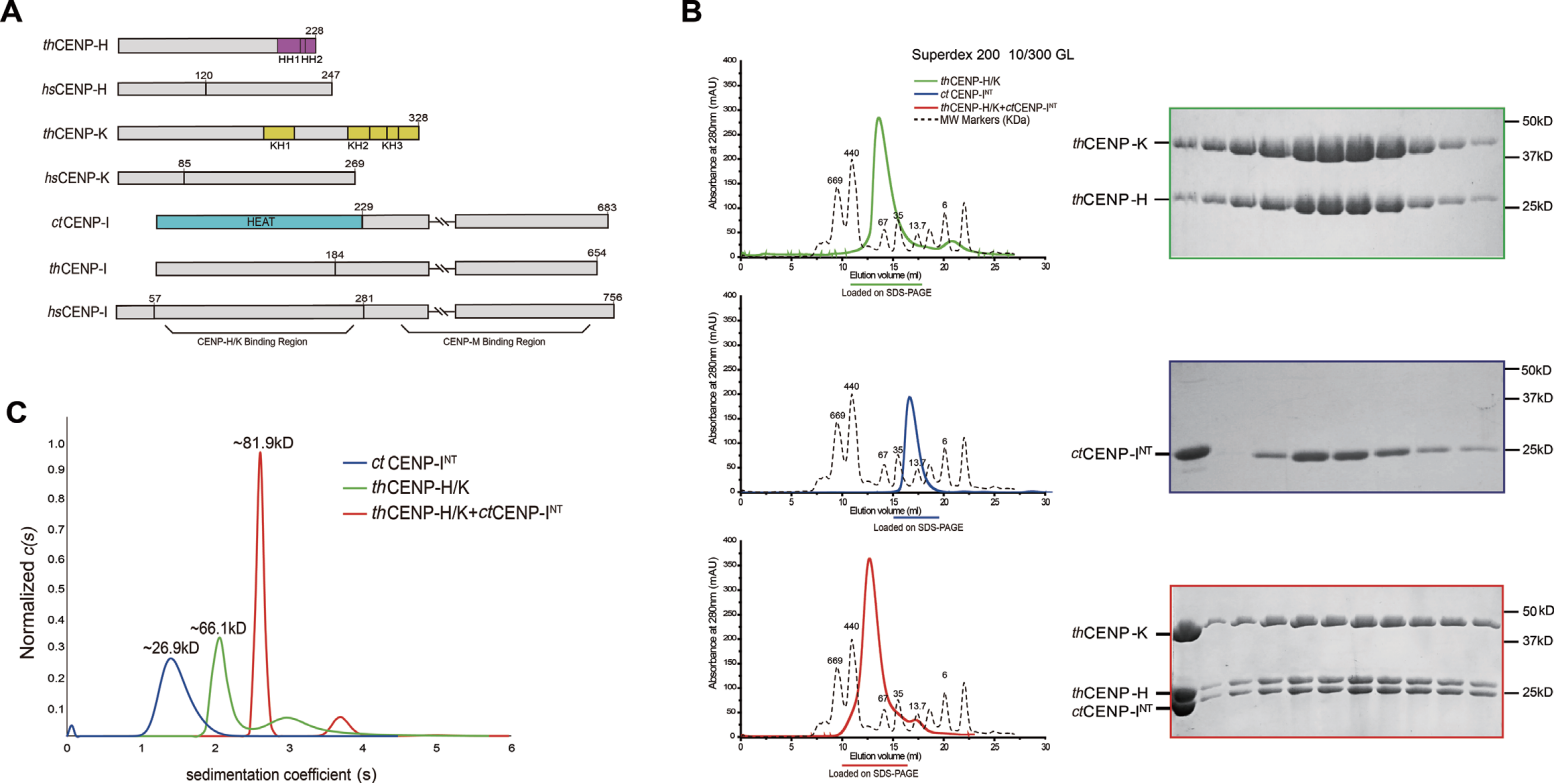
*In vitro* reconstitution of CENP-H/I/K complex from thermophilic fungi. (**A**) Schematic view of *th*CENP-H/I/K, *hs*CENP-H/I/K and *ct*CENP-I proteins. The colored regions denote the domains revealed in the structures. The numbered regions represent the constructs used in this study. It has been identified previously that N-terminus of *hs*CENP-I binds to CENP-H/K, and C-terminus interacts with CENP-M (39). (**B**) SEC elution profiles of *th*CENP-H/K, *ct*CENP-I^NT^ and ternary complex, respectively, from top to bottom. Peak fractions were resolved with SDS-PAGE and stained with Coomassie Blue. (**C**) SV AUC analysis of *ct*CENP-I^NT^ (blue), *th*CENP-H/K (green) and ternary complex (red). The molecular weights were calculated based on sedimentation coefficient, obtained with SEDFIT software using a continuous c(s) model.

Our attempts to reconstitute *Homo sapiens* (*hs*) CENP-H/I/K complex for structural analyses failed due to poor stability of *hs*CENP-I. We therefore searched for orthologs of these proteins from other species. Searches in the Pfam and InterPro databases showed that the protein G2R207, G2QRQ0 and G2R3T1 from *T. terrestris* (*th*) display significant sequence homology to human CENP-H, CENP-I and CENP-K, respectively (Figure 1A) (53,54). Reciprocal Best BLAST searches strongly support the orthologous relationship between fungi and human proteins. For example, the sequence of G2QRQ0 is significantly similar to *hs*CENP-I (40% similarity, *E*-value 7.8 × 10^−20^). We also constructed phylogenetic trees and sequence alignments of CENP-H, I and K from divergent species, which further demonstrated the homology relationship between fungi, yeast and human proteins (Supplementary Figures S1–4). Therefore, we annotated these three proteins in *T. terrestris* as CENP-H, CENP-I and CENP-K homologs (*th*CENP-H/I/K) (Figure 1A), respectively. The *th*CENP-H/I/K complex expressed at higher levels and showed better stability, but did not crystallize (Supplementary Figure S1D). Instead, we found that CENP-I from another fungi *C. thermophilum* (*ct*) is 62% identical in sequence to *th*CENP-I, and showed excellent biochemical behavior. However, we could not reconstitute the *ct*CENP-H/I/K complex because there is no CENP-H homolog identified in *C. thermophilum*.

Due to the high degree of sequence similarity, we tested whether *ct*CENP-I could form a hybrid complex with *th*CENP-H/K for crystallization. It has been shown that the N-terminal region of *hs*CENP-I is necessary and sufficient for interacting with *hs*CENP-H/K (32,39). We therefore purified the corresponding N-terminal region of *ct*CENP-I (*ct*CENP-I^NT^) (Figure 1A), which indeed formed a stable hybrid complex with the *th*CENP-H/K proteins (Figure 1B). The analytical ultracentrifugation (AUC) analyses showed that the hybrid CENP-H/I/K complex had a stoichiometry of 1:1:1 (Figure 1C).

**Figure 2.**
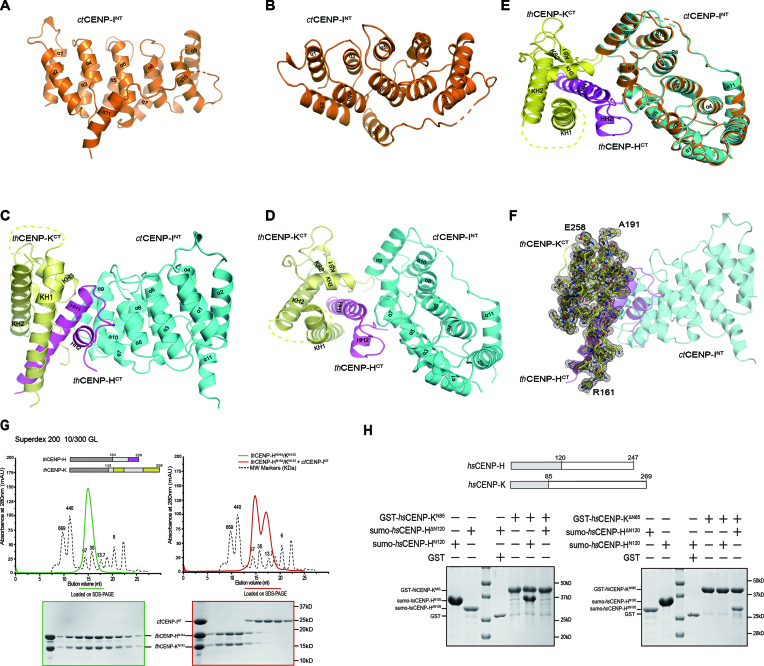
Crystal structures of *ct*CENP-I^NT^ alone and its complex with *th*CENP-H^CT^/K^CT^. (**A** and **B**) Cartoon view of the crystal structure of *ct*CENP-I^NT^ alone in side (A) and top (B) orientations. Residues 198–205 between α10 and α11 not traceable in the electron density map are shown as dotted lines. (**C** and **D**) Cartoon view of the crystal structure of fungal CENP-H/I/K complex in side (C) and top (D) orientations. The *th*CENP-K^CT^, *th*CENP-H^CT^ and *ct*CENP-I^NT^ are colored yellow, magenta and cyan, respectively. ( **E**) Electron density map for *th*CENP-K^CT^ in the complex structure. The composite omit map was calculated with Phenix, contoured at 1.0σ. There is no electron density for residues 192–267 between KH1 and KH2 of *th*CENP-K. (**F**) Overlay of the ribbon diagrams of the *ct*CENP-I^NT^ alone (orange) and its complex with *th*CENP-H^CT^/K^CT^ (colored yellow, magenta and cyan, respectively). (**G**) SEC elution profiles of *th*CENP-H^N164^/CENP-K^N143^ complex (left) and its mixture with *ct*CENP-I^NT^ (right). Peak fractions were resolved with SDS-PAGE and stained with Coomassie Blue. (**H**) The N-termini of *hs*CENP-K only bound to the N-terminal fragment of *hs*CENP-H (left panel), the C-termini of *hs*CENP-K only bound to C-terminal fragment of *hs*CENP-H (right panel). GST-*hs*CENP-K^N85^ and GST-*hs*CENP-K^ΔN85^ were immobilized on beads and incubated with sumo-*hs*CENP-H^N120^ or sumo-*hs*CENP-H^ΔN120^. The bead-bound proteins were resolved with SDS-PAGE and stained with Coomassie Blue.

We first determined the structure of *ct*CENP-I^NT^ at 2.3Å with the Single-wavelength Anomalous Dispersion (SAD) method (Supplementary Table S1). *ct*CENP-I^NT^ adopts the HEAT repeat fold containing five pairs of helixes α1–α10. A following helix α11 folds back and packs against the HEAT repeat with extensive hydrophobic interactions, which likely contributes to the protein stability (Figure 2A and B). The structure of *ct*CENP-I is similar to a model of *hs*CENP-I proposed previously (39), except that helix α11 packs on the surface of the HEAT repeat in our structure.

We also determined the crystal structure of the hybrid complex between *ct*CENP-I^NT^ and *th*CENP-H/K at 2.2Å with the Molecular Replacement method using the *ct*CENP-I^NT^ structure as start model (Figure 2C and D; Supplementary Table S1). The structure of *ct*CENP-I^NT^ in the complex is very similar to the apo state (Figure 2E). In one asymmetric unit of the complex crystal, one *th*CENP-H/K heterodimer interacts with one *ct*CENP-I^NT^ homodimer (Supplementary Figure S5A). The homodimer of *ct*CENP-I^NT^ might be a crystal packing artifact, since the complex shows a 1:1:1 stoichiometry in solution (Figure 1C). Although the full-length *th*CENP-H/K proteins were used in crystallization, only the C-terminal portions of *th*CENP-H (184–228aa, *th*CENP-H^CT^) and *th*CENP-K (161–328aa, *th*CENP-K^CT^) are found in the structure (Figure 2C and D). The N-terminal portions of *th*CENP-H and *th*CENP-K are missing in the electron density map, which might be caused by protein degradation during crystallization, as shorter fragments were observed in electrophoresis analyses of the complex crystals (Supplementary Figure S5B).

Overall, the ternary complex adopts a sandwich-like structure. *ct*CENP-I^NT^ and *th*CENP-K^CT^ on each side pack against *th*CENP-H^CT^ in the middle (Figure 2C and D). *th*CENP-H^CT^ contains two α-helixes HH1 and HH2 to interact with both *th*CENP-K^CT^ and *ct*CENP-I^NT^. In the structure of *th*CENP-K^CT^, three helixes KH1, KH2 and KH3 wrap around the HH1 helix of *th*CENP-H^CT^ (Figure 2C and D). The loop region (192–257aa) between KH1 and KH2 is invisible in the electron density map (Figure 2F). This loop region is not conserved in *hs*CENP-K, suggesting that it may not be functional important (Supplementary Figure S3). On the other side of the complex, helixes α5 and α7 of *ct*CENP-I^NT^ contact with HH2 helix of *th*CENP-H^CT^ (Figure 2C and D).

### CENP-H and CENP-K form a heterodimer through both N-terminal and C-terminal interactions

To clarify the role of the N-terminal portions of *th*CENP-H and *th*CENP-K, which are missing in the structure, we purified C-terminal truncated version of *th*CENP-H and *th*CENP-K. We found that *th*CENP-H^N164^ and *th*CENP-K^N143^ form a stable complex in gel-filtration chromatography (Figure 2G, left panel). These results indicated that *th*CENP-H and *th*CENP-K form a heterodimer through both N-terminal and C-terminal interaction. Addition of *ct*CENP-I^NT^ did not alter the behavior of *th*CENP-H^N164^/K^N143^ complex in the gel-filtration analysis (Figure 2G, right panel). Consistently, no obvious interaction between *ct*CENP-I^NT^ and *th*CENP-H^N164^/K^N143^ was observed in GST pull-down experiments (Supplementary Figure S6A). These results together confirmed that *ct*CENP-I^NT^ only interact with the C-termini of *th*CENP-H/K complex as shown in the crystal structure.

To establish the generality of this dual binding mode in CENP-H/K complex, we also characterized the interactions between human CENP-H and CENP-K using GST pull-down assay. The results showed that the N-terminal fragment of *hs*CENP-K (*hs*CENP-K^N85^) only bound to the N-terminal portion of *hs*CENP-H (*hs*CENP-H^N120^) (Figure 2H, left panel), whereas the C-terminal fragment of *hs*CENP-K (*hs*CENP-K^ΔN85^) only bound to C-terminal portion of *hs*CENP-H (*hs*CENP-H^ΔN120^) (Figure 2H, right panel). Thus, the dual binding model represents a conserved mechanism for the formation of the CENP-H/K complex.

### Conserved residues mediate C-terminal interaction of CENP-H/K complex

**Figure 3.**
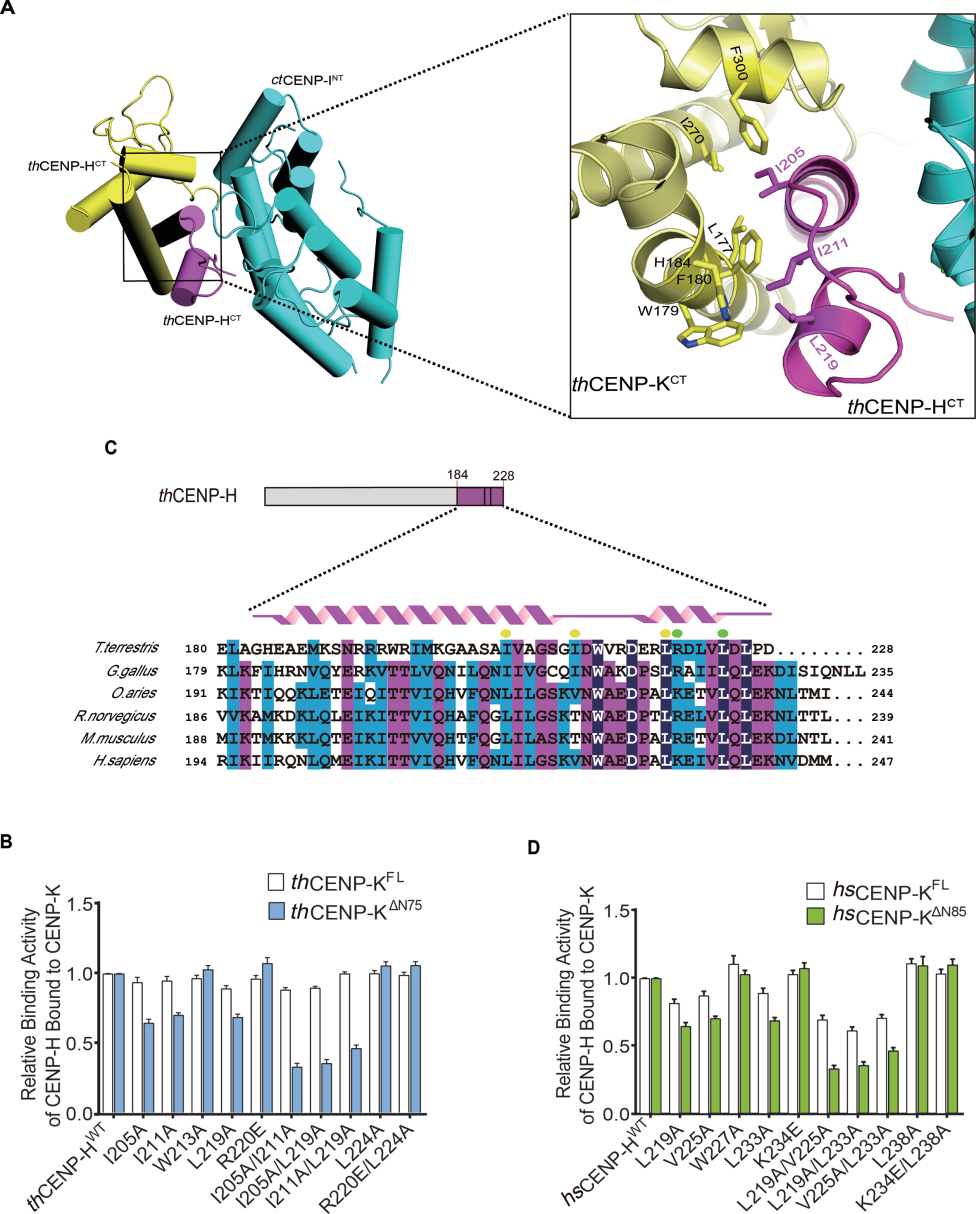
Validate the C-terminal binding interface between CENP-H and CENP-K. (**A**) Zoomed-in view of the binding interface between *th*CENP-H^CT^ and *th*CENP-K^CT^ in the crystal structure of ternary complex. The interacting residues were drawn in stick and marked in number. (**B**) The relative binding activity of *th*CENP-H (WT and mutants) bound to GST-*th*CENP-K^FL^ and GST-*th*CENP-K^ΔN75^ was assessed using GST pull-down assays. Error bars represent standard deviations, which were obtained from three independent experiments. The representative results of the pull-down assays were also available in Supplementary Figure S6B and C. (**C**) Alignment of the C-terminus sequences of CENP-H orthologs across species using Clustal Omega Program. The conserved residues were colored. The interacting residues identified in our structures were marked with yellow dots (binding to *th*CENP-K^CT^) and green dots (binding to *ct*CENP-I^NT^). ( **D**) The relative binding activity of *hs*CENP-H (WT and mutants) bound to GST-*hs*CENP-K^FL^ and GST-*hs*CENP-K^ΔN85^ was assessed using GST pull-down assays. Error bars represent standard deviations, which were obtained from three independent experiments. The representative results of the pull-down assays were also available in Supplementary Figure S7A and B.

The structure of fungal CENP-H/I/K complex revealed the details of the C-terminal binding interface of CENP-H/K complex. The sidechain of *th*CENP-H I205 inserts into a hydrophobic cavity surrounded by L177, F180, I270 and F300 of *th*CENP-K. On the other side, I211 and L219 of *th*CENP-H contact with the hydrophobic patch formed by W179, F180 and H184 of *th*CENP-K (Figure 3A). To validate these interactions, we mutated the interface residues in *th*CENP-H and tested the effects of these mutations in an *in vitro* binding assay. The relative binding activity was measured by the amount of bound *th*CENP-H normalized to that of GST-tagged protein on the GST beads. We found that *th*CENP-H WT robustly bound to GST-*th*CENP-K^ΔN75^. The single mutants *th*CENP-H I205A, I211A and L219A, exhibited weaker binding to *th*CENP-K^ΔN75^. As expected, the weakened effects of the double mutants I205A/I211A and I205A/L219A were much stronger (blue column in Figure 3B and Supplementary Figure S6B). In contrast, mutations of the neighboring residues, such as R220 or L224, did not alter the binding to *th*CENP-K^ΔN75^ (Figure 3B). Thus, the C-terminal surface revealed by our crystal structure indeed mediates the interaction of *th*CENP-H/K complex. Interestingly, the full-length *th*CENP-K (*th*CENP-K^FL^) retained binding to the *th*CENP-H mutants (blank column in Figure 3B and Supplementary Figure S6C). For example, *th*CENP-H I205A/L219A significantly weakened the binding to *th*CENP-K^ΔN75^, but remained the binding to *th*CENP-K^FL^ (Figure 3B). These results further confirmed the N-terminal interaction between *th*CENP-H and *th*CENP-K.

The sequence alignment of CENP-H orthologs showed that the residues involved in binding CENP-K are highly conserved (Figure 3C). We mutated the corresponding residues in *hs*CENP-H, including L219, V225 and L233 (yellow spots in Figure 3C), and tested the binding to *hs*CENP-K full length (*hs*CENP-K^FL^) or N-terminal truncation (*hs*CENP-K^ΔN85^) in the GST pull-down assay (Figure 3D; Supplementary Figure S7A and B). The results are similar to those with *th*CENP-H and *th*CENP-K. For instance, *hs*CENP-H L219A/L233A lost the interaction with *hs*CENP-K^ΔN85^, but retained partial binding to *hs*CENP-K^FL^ (Figure 3D). These results suggest the binding mode between CENP-H and CENP-K as shown by our crystal structure are conserved in human.

### CENP-H directly interacts with CENP-I through the conserved C-terminal surface

**Figure 4.**
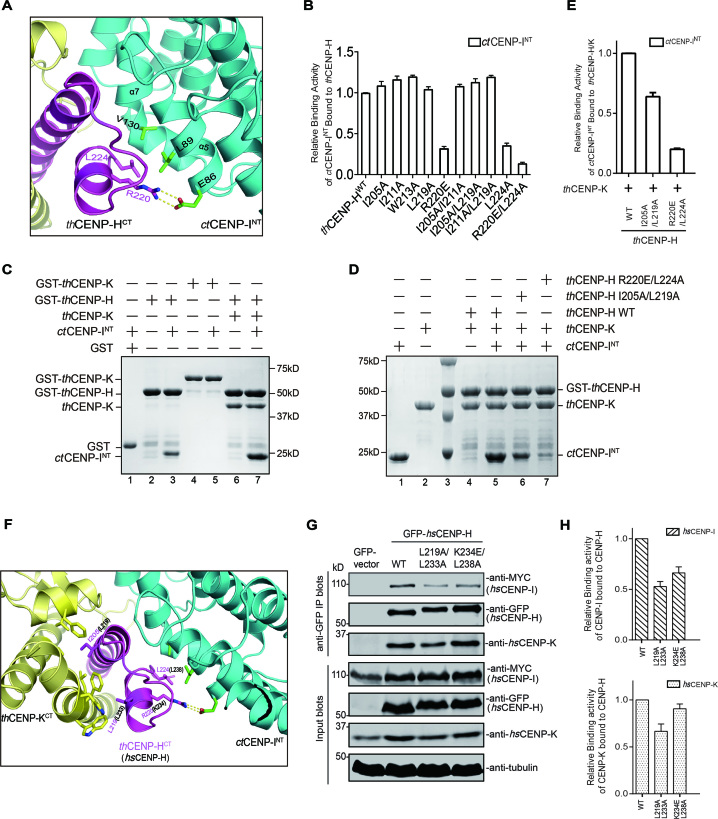
Validate the binding interface between CENP-H and CENP-I, and the assembly mode of CENP-H/I/K ternary complex. ( **A**) Zoomed-in view of the binding interface between *th*CENP-H^CT^ and *ct*CENP-I^NT^. The interacting residues were drawn in stick and marked in number. (**B**) The relative binding activity of *th*CENP-H (WT and mutants) bound to GST-*ct*CENP-I^NT^ was assessed using GST pull-down assays. Error bars represent standard deviations, which were obtained from three independent experiments. The representative results of the pull-down assays were also available in Supplementary Figure S8A. (**C**) *In vitro* binding assays were performed to examine the binding activity among the ternary complex. Proteins with distinct combinations as indicated were mixed and GST pull-down assays were performed. The bead-bound proteins were resolved with SDS-PAGE and stained with Coomassie Blue. (**D**) *In vitro* binding assays were performed to assessing the effects of *th*CENP-H mutants on the formation of the fungal CENP-H/I/K ternary complex. Proteins with distinct combinations as indicated were mixed and GST pull-down assays were performed. The bead-bound proteins were resolved with SDS-PAGE and stained with Coomassie Blue. (**E**) Quantification of the relative binding activity between *ct*CENP-I^NT^ and *th*CENP-H (WT and mutants) in the presence of *th*CENP-K based on the results from lanes 5, 6 and 7 in (D). (**F**) Zoomed-in view of the binding interfaces of *th*CENP-H^CT^ in the complex structure. The interacting resides in *th*CENP-H were drawn in stick and marked with numbers in red. And the counterpart residues in *hs*CENP-H were shown with numbers in black. ( **G**) HeLa Tet-On cells transfected with GFP-*hs*CENP-H (WT or mutants), non-tagged *hs*CENP-K and MYC-*hs*CENP-I. Cell lysates were treated with anti-GFP antibody. Immunoprecipitated samples were resolved with SDS-PAGE and blotted with the indicated antibodies. ( **H**) Quantification of the relative binding activity between *hs*CENP-I and *hs*CENP-H WT or mutants (upper panel), and between *hs*CENP-K and *hs*CENP-H WT or mutants (lower panel) based on the results from Figure 4G.

*th*CENP-H mainly utilizes its HH2 helix to interact with the HEAT repeat surface of *ct*CENP-I^NT^. The salt-bridge is formed between R220 in *th*CENP-H and E86 of *ct*CENP-I^NT^. L224 in *th*CENP-H interacts with the hydrophobic concavity formed by L89, V126 and V130 in *ct*CENP-I^NT^ (Figure 4A). To validate these interactions, we mutated the interface residues in *th*CENP-H and tested the binding activity. The results showed that the R220E and L224A mutations of *th*CENP-H dramatically decreased the binding to GST-*ct*CENP-I^NT^, while the R220E/L224A double mutation completely abolished the binding (Figure 4B and Supplementary Figure S8A). Thus, the binding interface revealed by our structure is critical for the interaction between *th*CENP-H and *ct*CENP-I^NT^. Furthermore, the sequence alignment of CENP-H clearly showed that K234 and L238 in *hs*CENP-H correspond to R220 and L224 in *th*CENP-H (Figure 3C), which are critical for binding to *ct*CENP-I, indicating a potential conserved interaction mode between CENP-H and CENP-I.

### CENP-K enhances the interaction between CENP-H and CENP-I to form the ternary complex

We next tested how these three proteins form ternary complex using *in vitro* pull-down assays. We found that *th*CENP-H can bind to *ct*CENP-I^NT^ and *th*CENP-K individually (lanes 3 and 6, Figure 4C), consistent with the structure of the complex in which *th*CENP-H is placed at the center and make extensive interactions with both *th*CENP-K and *ct*CENP-I^NT^. In contrast, *th*CENP-K did not directly interact with *ct*CENP-I^NT^ (lane 5, Figure 4C). Interestingly, addition of *th*CENP-K significantly increased the binding of *ct*CENP-I^NT^ to *th*CENP-H (lanes 3 and 7 in Figure 4C). The enhancement effect of *th*CENP-K is lost for the *th*CENP-H I205A/L219A (Figure 4D and E), which disrupts the C-terminal interaction of *th*CENP-H/K (Figure 3B). In contrast, *ct*CENP-I^NT^ did not affect the interactions between *th*CENP-H and *th*CENP-K (Figure 4C), and the *th*CENP-H R220E/L224A also disrupted the binding of *ct*CENP-I^NT^ without affecting the *th*CENP-H/K interactions (Figure 4D and E). These results together suggest that formation of CENP-H/K complex enhances the recruitment of CENP-I. It is possible that binding of *th*CENP-K to *th*CENP-H might induce conformational changes in *th*CENP-H, thus presenting a better position of R220 and L224 for *ct*CENP-I^NT^ binding (Figure 4F).

We then asked whether *hs*CENP-K can also promote the assembly of the human CENP-H/I/K complex as *th*CENP-K does. The sequence alignment of CENP-H showed that K234/L238 in *hs*CENP-H correspond to R220/L224 in *th*CENP-H for *ct*CENP-I^NT^ binding, while L219/L233 in *hs*CENP-H correspond to I205/L219 in *th*CENP-H for *th*CENP-K binding (Figures 3C and 4F). We mutated these two potential interfaces in *hs*CENP-H to test the assembly of CENP-H/I/K ternary complex in HeLa cells. HeLa cells were co-transfected with MYC-*hs*CENP-I, non-tagged *hs*CENP-K and GFP-*hs*CENP-H WT or mutants. The formation of CENP-H/I/K complex was assessed by immunoprecipitation experiments using an anti-GFP antibody. The results showed that GFP-*hs*CENP-H WT robustly bound to both *hs*CENP-I and *hs*CENP-K (Figure 4G). As expected, GFP-*hs*CENP-H K234E/L238A showed a diminished binding to *hs*CENP-I, but retained the ability to bind *hs*CENP-K (Figure 4G and H), which is consistent with the binding results of fungal protein (Figure 4B and D). These results strongly support the notion that the binding interface between CENP-H and CENP-I are conserved from fungi to human. Interestingly, GFP-*hs*CENP-H L219A/L233A not only exhibited a decreased binding to *hs*CENP-K, also exhibited a significantly decreased binding to *hs*CENP-I (Figure 4G and H), as *th*CENP-H I205A/L219A did to *th*CENP-I^NT^ for the ternary complex formation (Figure 4D and E). These results highlighted that the enhancement of CENP-K is also critical for human CENP-H/I/K complex formation. Thus, all these results indicated that human CENP-H/I/K complex likely adopts a similar interaction mode as its thermophile fungus orthologs, suggesting that this assembly mode may be conserved across species.

### Maintaining the proper assembly of CENP-H/I/K complex is essential for centromeric localization and faithful chromosome segregation

**Figure 5.**
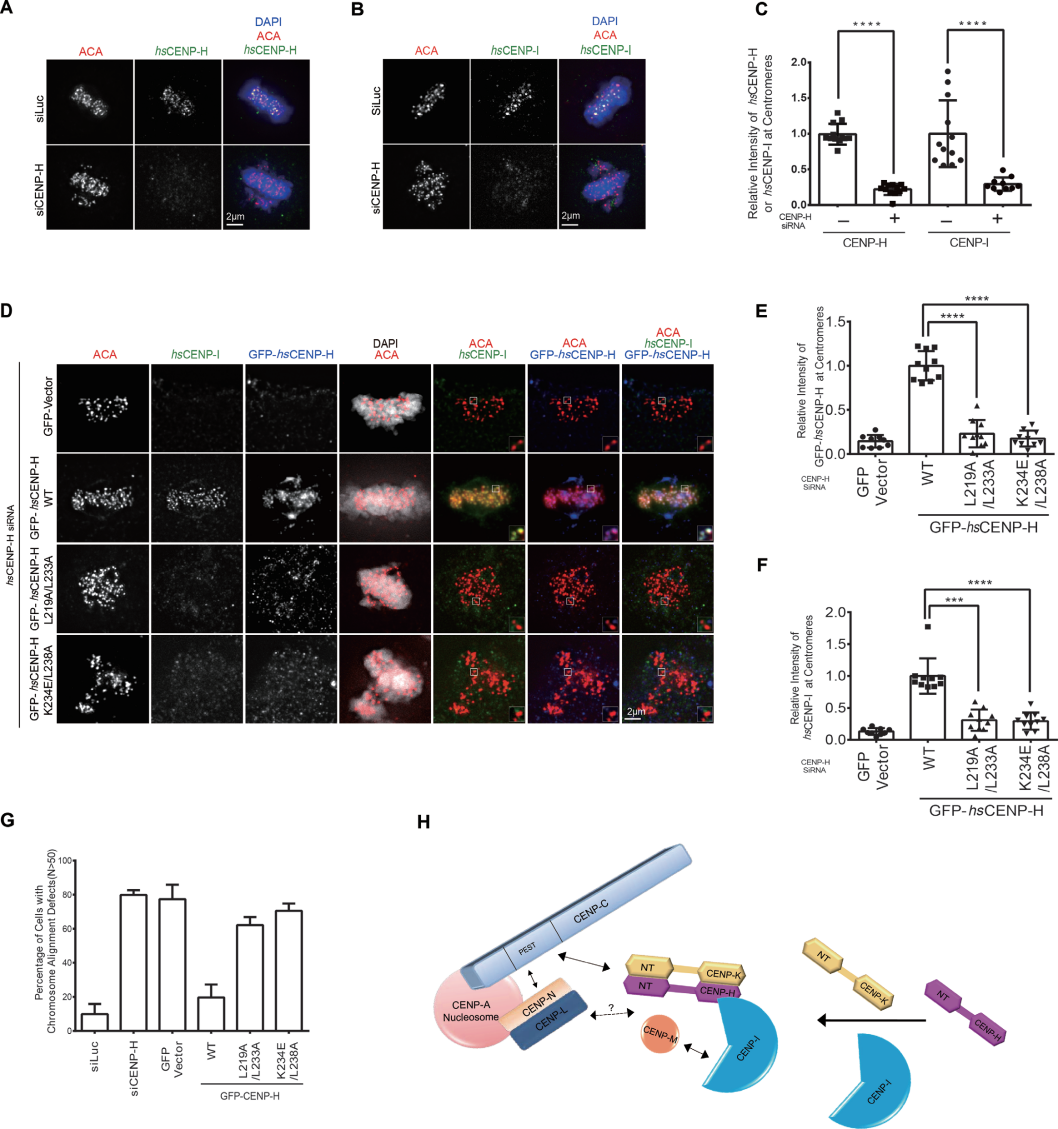
Mutating the interface residues of CENP-H abolished its centromeric localization and induced massive chromosome mis-alignment. (**A** and **B**) Representative images of interphase HeLa Tet-On cells treated with Luciferase and *hs*CENP-H siRNA. The centromeric localization of endogenous *hs*CENP-H (A) or *hs*CENP-I (B) was detected by indicated antibody. (**C**) Quantification of the centromeric intensities of *hs*CENP-H in (A) and *hs*CENP-I in (B) normalized to the ones of ACA (CREST). At least 10 cells (20 kinetochores per cell) were quantified for each condition. Mean ± SD (standard deviation) was shown here. (**D**) HeLa Tet-On cells transfected with RNAi-resistant GFP-*hs*CENP-I WT, L219AL233A and K234E/L238A were treated with *hs*CENP-H siRNA. Cells were briefly treated with MG132 before subjected to staining. Representative images of mitotic cells were shown here. (**E** and **F**) Quantification of the centromeric intensities of GFP-*hs*CENP-H (E) and *hs*CENP-I (F) normalized to the ones of ACA (CREST) kinetochore signal. At least 10 cells (20 kinetochores per cell) were quantified for each condition. Mean ± SD was shown here. (**G**) Quantification of mitotic cells with chromosome alignment defects for the experiments in (A, B and D). At least 50 cells were counted for each condition. Mean ± SD was shown here. (**H**) The assembly mode of CENP-H/I/K ternary complex among the CCAN. CENP-A directly recruits CENP-C and CENP-L/N to the centromere (25,26,29,32,43,44). Both CENP-C and CENP-L/N are required for kinetochore recruitment of CENP-/H//I/K/M (32,43). CENP-C directly interacts with CENP-H/K, not CENP-I (32). How CENP-L/N interacts with CENP-H/I/K/M remains unclear. CENP-H/K form a heterodimer through both N-termini and C-termini, and the C-termini of CENP-H/K heterodimer binds to N-terminus of CENP-I. CENP-M directly interacts with the C-terminus of CENP-I and integrate into the CENP-H/I/K/M complex (39).

We also examined the centromeric localization of *hs*CENP-H and *hs*CENP-I in mitotic cells. Both *hs*CENP-H and *hs*CENP-I co-localized with the centromere marker CREST in the control cells (Figure 5A and B). Depletion of *hs*CENP-H by treatment with *hs*CENP-H siRNA almost completely abolished the centromeric localizations of both *hs*CENP-H and *hs*CENP-I (Figure 5A–C). This was not caused by cross effects of the siRNA since *hs*CENP-H siRNA significantly decreased the protein level of *hs*CENP-H, but not *hs*CENP-I, and vice versa (Supplementary Figure S8B).

We next sought to investigate whether the interactions among the CENP-H/I/K complex affect their centromeric localization during mitosis. siRNA-resistant GFP-*hs*CENP-H WT expressed in *hs*CENP-H depleted cells, showed the normal centromeric localization and restored the localizations of *hs*CENP-I to centromeres (Figure 5D–F). In contrast, both *hs*CENP-H L219A/L233A and K234E/L238A failed to localize into centromeres, and therefore could not restore *hs*CENP-I localization (Figure 5D–F). The localization defects of *hs*CENP-H mutants was unlikely caused by decreased expression levels or protein stability, since they were expressed at levels comparable to the wild- type protein and interacted with *hs*CENP-K (Supplementary Figure S8C and Figure 4G). Thus, these results suggested that the interactions among CENP-H/I/K complex are essential for maintaining their centromeric localizations during mitosis, and confirmed previous reports showing that centeromeric localization of these proteins are inter-dependent (12,13,16,43,47,48). Therefore, efficient incorporation of these proteins into the kinetochore requires the proper formation of the CENP-H/I/K ternary complex.

Moreover, we examined the chromosome alignment phenotype during mitosis. Approximately 80% of *hs*CENP-H depleted cells (*n* > 50) exhibited significant chromosome alignment defects, while chromosomes aligned properly in most of the control cells (Figure 5A, B and G). The expression of GFP-*hs*CENP-H WT significantly rescued the chromosome alignment defects in *hs*CENP-H depleted cells (Figure 5D and G). In contrast, expressions of the *hs*CENP-H L219A/L233A and K234E/L238A mutants failed to do so (Figure 5D and G). These results show that the interactions among the CENP-H/I/K complex are critical for proper chromosome alignment and segregation in mitosis.

## DISCUSSION

Determining how the CCAN subunits are organized is important for understanding the kinetochore assembly and function. Among the CCAN subunits, CENP-H, CENP-I, CENP-K and CENP-M have been previously shown to form a stable sub-complex based on proteomic analyses and reconstitution experiments (12,13,16,24,30,35,36, 39). In addition, a previous study has presented the low-resolution structure and model of the CENP-H/I/K/M quaternary complex (39). Our study for the first time provides the high-resolution structure of the fugal CENP-H/I/K complex. Further analyses suggest that the interactions revealed in this complex represent the evolutionarily conserved mechanism for the assembly of the CENP-H/I/K complex.

CENP-A nucleosome directly recruits CENP-C and CENP-L/N to the centromere, and initiates assembly of the CCAN (25,26,29,32,43,44). CENP-C has been shown to directly interact with CENP-H/K, not CENP-I, which contributes to centromeric recruitment of the CENP-H/I/K/M complex (32). CENP-L/N and CENP-H/I/K/M are mutually required for centromeric localization (43), although the detailed interactions remain unclear. CENP-M directly interact with the C-terminus of CENP-I and integrate into the CENP-H/I/K/M complex (39). Our findings demonstrate that CENP-H/K form a heterodimer through both N-termini and C-termini, and the C-termini of CENP-H/K heterodimer binds to the N-terminus of CENP-I (Figure 5H). The roles of the central regions of CENP-H/K complex are currently unclear. They might be involved in the ternary complex formation or interacting with other CCAN components. In comparison with the previously co-linear interaction model based on the low-resolution structure (39), our results suggest a slightly distinct model explaining how the CENP-H/I/K complex is assembled. The difference might be due to divergent experimental conditions or species difference. Alternatively, it might reflect a possibility that the CENP-H/I/K complex exists in distinct conformations in cells.

Although loss of each component of the CENP-H/I/K complex has been shown to cause chromosome mis-segregation (12,13,16,43,47,48), the exact roles of the individual protein–protein interfaces in chromosome segregation had never been investigated. Our results indicate that any flaw in these interactions can result in massive chromosome mis-alignment, suggestive of importance of the integrity of the CENP-H/I/K complex in proper kinetochore function. The CENP-H/I/K/M complex has also been shown to interact with CENP-T/W and CENP-L/N (32,43). In future, it will be important to identify the interacting surfaces and evaluate their functional importance in kinetochore function.

## DATA AVAILABILITY

Structures and crystallographic data have been deposited at the wwPDB: 5Z07 (*ct*CENP-I^NT^) and 5Z08 (*ct*CENP-I^NT^ complex with *th*CENP-H^CT^/K^CT^).

## Supplementary Material

Supplementary Data
