# Supplementary material for: Structural analysis of fungal CENP-H/I/K homologs reveals a conserved assembly mechanism underlying proper chromosome alignment
Source: Nucleic Acids Res. 2018 Nov 8;47(1):468–79. doi: 10.1093/nar/gky1108 (PMC6326798; doi:10.1093/nar/gky1108)
Supplement: Supplementary Data [file gky1108_supplemental_files.pdf]

SUPPLEMENTARY INFORMATION

Structural Analysis of Fungi CENP-H/I/K Complex Reveals its Conserved Assembly Mechanism Underlying Faithful Chromosome Alignment

Liqiao Hu[1, †], Hao Huang[1, †], Mohan Hei[1], Yang Yang[2], Sheng Li[3], Yunshan Liu[1], Zhen Dou[4], Mengying Wu[1], Jie Li[5], Guang-zhong Wang[5], Xuebiao Yao[4], Hong Liu[6], Xiaojing He[1, *], Wei Tian[1, *]

[1]Key Laboratory of Molecular Biophysics of the Ministry of Education, College of Life Science and Technology, Huazhong University of Science and Technology, Wuhan 430074, China

[2]Department of Pharmacology, University of Texas Southwestern Medical Center, Dallas, TX 75390, USA

[3]Shanghai Institute for Advanced Immunochemical Studies, ShanghaiTech University, Shanghai 201210, China

[4]Anhui Key Laboratory for Cellular Dynamics & Chemical Biology, Hefei National Research Center for Physical Sciences at the Microscale, University of Science & Technology of China, Hefei 230027, China

[5]CAS Key Laboratory of Computational Biology, CAS-MPG Partner Institute for Computational Biology, Shanghai Institute of Nutrition and Health, Shanghai Institutes for Biological Sciences, University of Chinese Academy of Sciences, Chinese Academy of Sciences, Shanghai 200031, China

[6]Department of Biochemistry and Molecular Biology, Tulane University Health Science Center, New Orleans, LA 70112, USA

[†]These authors contributed equally to this work.

*To whom correspondence may be addressed. Email: hexj@hust.edu.cn; or tianwei@hust.edu.cn

SUPPLEMENTAL TABLE AND FIGURE LEGENDS

Table S1. Data collection, phasing and refinement statistics for crystal structure of *ct*CENP-I^{NT} alone and *ct*CENP-I^{NT} complex with *th*CENP-H^{CT}/K^{CT}

Figure S1. The phylogenetic tree of CENP-I (A), CENP-H (B) and CENP-K (C) from divergent species, and the reconstitution of thCENP-H/I^{NT}/K complex (D).

Figure S2. Sequence alignment of the CENP-I from multiple species
The alignment is generated using the online ESPript 3.0 server. Secondary structural elements predicted from PSI-PRED online server and found from crystal structure are indicated above the sequences, with the color blue and gray. The sequence of ctCENP-I shares high similarity (50%) with human CENP-I (E-value 7.65E-20) and high identity (62%) with thCENP-I.

Figure. S3 Sequence alignment of the CENP-K from multiple species
The sequence similarity between thCENP-K and hsCENP-K is 40%, based on the reciprocal Best BLAST result (E-value= 1.81E-06).

Figure. S4 Sequence alignment of the CENP-H from multiple species.
The thCENP-H exhibits a relative low sequence similarity (29%) with hsCENP-H, but the residues in involved the binding interfaces for the complex formation show higher conservation levels.

Figure S5. Density maps of *th*CENP-H^{CT} and *th*CENP-K^{CT}, and crystal structure of the ternary complex in one asymmetric unit
A. Cartoon view of fungi CENP-H/I/K complex structure. Even though two molecules of *ct*CENP-I^{NT} (cyan and green) were found in one asymmetric unit, only one binds to the heterodimer of *th*CENP-H^{CT} (magenta) and *th*CENP-K^{CT} (yellow).
B. SDS-PAGE electrophoresis analyses of the complex crystals, stained with Coomassie Blue.
C. Electron density for *th*CENP-H^{CT} in the complex structure, composite omit map calculated with Phenix, contoured at 1.0σ.

Figure S6. Analyses of the binding activities of *th*CENP-H WT or mutants bound to GST-*th*CENP-K$^{\Delta N75}$ and GST-*th*CENP-K^{FL}

A. *In vitro* binding assay were performed to examine the binding activity between thCENP-H^{N164} (ΔCT) and thCENP-K^{N143} (ΔCT) complex and ctCENP-I^{NT}.

B and **C.** Recombinant *th*CENP-H WT or mutant proteins were mixed with GST or GST-*th*CENP-K$^{\Delta N75}$ (**B**) and GST-*th*CENP-K^{FL} (**C**) proteins**.** GST pull-down assays were performed. The bead-bound proteins were resolved with SDS-PAGE and stained with Coomassie Blue. Quantification was summarized for both in **Figure 3B**.

Figure S7. Analyses of the binding activities of *hs*CENP-H WT or mutants bound to GST-*hs*CENP-K$^{\Delta N85}$ and GST-*hs*CENP-K^{FL}

A and **B.** Recombinant *hs*CENP-H WT or mutant proteins were mixed with GST or GST-*hs*CENP-K$^{\Delta N85}$ (**A**) and GST-*hs*CENP-K^{FL} (**B**) proteins**.** GST pull-down assays were performed. The bead-bound proteins were resolved with SDS-PAGE and stained with Coomassie Blue. Quantification was summarized for both (**A** and **B**) in **Figure 3D**.

Figure S8. Analyses of the binding activities of *th*CENP-H WT or mutants bound to *ct*CENP-I^{NT}, and Immuno-blotting analyses of gene knock-down and overexpression in cell experiment.

A. Recombinant *th*CENP-H WT or mutant proteins were mixed with GST or GST-*ct*CENP-I^{NT} proteins. GST pull-down assays were performed. The bead-bound proteins were resolved with SDS-PAGE and stained with Coomassie Blue. Quantification was available in **Figure 4B**.

B. Cell lysates of HeLa Tet-On cells treated with Luciferase, *hs*CENP-H or *hs*CENP-I siRNA were resolved with SDS-PAGE and blotted with the indicated antibodies.

C. Cell lysates of HeLa Tet-On cells in **Figure 5D** were resolved with SDS-PAGE and blotted with the indicated antibodies.

Data collection, phasing and refinement statistics

	*ct*CENP-I^{NT}(Se-Met)	*ct*CENP-I^{NT} complex with *th*CENP-H^{CT}/ K^{CT}
**Wavelength**	0.9778	0.986
**Resolution range (Å)**	46.197 - 2.298(2.38-2.30)*	46.46 - 2.199 (2.24-2.20)*
**Space group**	P 41212	P 41
**Unit cell (a, b, c (Å))**	59.671 59.671 145.971 90 90 90	67.847 67.847 186.269 90 90 90
**Unique reflections**	22388(2244)	42600 (4220)
**Multiplicity**	4.5 (4.5)	9.4 (7.6)
**Completeness (%)**	99.7 (99.9)	100 (99.5)
**Mean I/sigma(I)**	21.42(3.25)	29.64(1.44)
**Wilson B-factor**	47.39	46.37
**R-meas**†	0.070(0.588)	0.102 (1.562)
**R-pim**‡	0.033 (0.274)	0.033 (0.559)
**$CC_{1/2}$**§	0.866	0.694
**Phase determination**		
**Anomalous scatterer**	Selenium (1 of 1 possible sites)	
**Figure of merit, 46.20–2.30 Å**	0.246	
**Refinement statistics**		
**Reflections used in refinement**	12379(1202)	42479 (2611)
**Reflections used for R-free**	1238 (134)	2143 (228)
**R-work**	0.1739 (0.2132)	0.1877 (0.3134)
**R-free**	0.2277 (0.2867)	0.2298 (0.3549)
**Number of non-hydrogen atoms**	1790	4699
**macromolecules**	1700	4579
**solvent**	90	120
**Protein residues**	213	544
**RMS(bonds)**	0.007	0.008
**RMS(angles)**	0.961	0.901
**Ramachandran favored (%)**#	98.09	97.14
**Ramachandran allowed (%)**#	1.91	2.5
**Ramachandran outliers (%)**#	0	0.36
**Rotamer outliers (%)**	3.24	0.82
**Clashscore**	4.03	8.82
**Average B-factor**	54.73	69.44
**macromolecules**	54.44	69.79
**solvent**	60.28	56.11

*Data for the highest-resolution shell are shown in parentheses

†R-meas = $\Sigma_h (n/n-1)^{1/2} \Sigma_i |I_i(h) - \langle I(h) \rangle| / \Sigma_h \Sigma_i I_i(h)$, where $I_i(h)$ and $\langle I(h) \rangle$ are the ith and mean measurement of the intensity of reflection h.

‡R-pim = 100 $\Sigma_h \Sigma_i [1/(n_h-1)]^{1/2} |I_{h,i} - \langle I_h \rangle / \Sigma_h \Sigma_i \langle I_{h,i} \rangle$, where n_h is the number of observations of reflections h.

§$CC_{1/2}$ values shown are for the highest resolution shell.

#As defined by the validation suite MolProbity.

Table S1

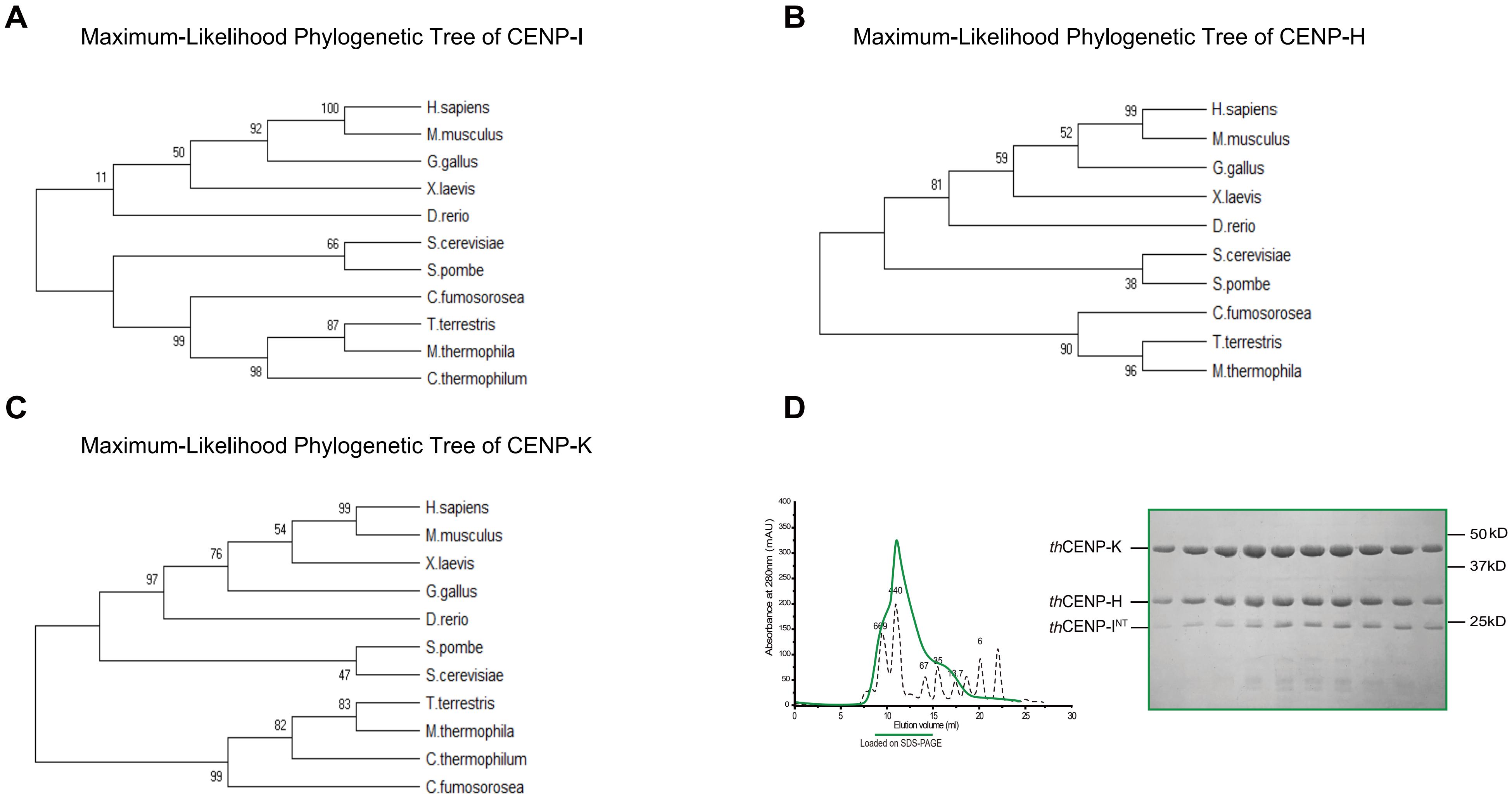
A
Maximum-Likelihood Phylogenetic Tree of CENP-I
100
92
50
11
66
87
99
98
H.sapiens
M.musculus
G.gallus
X.laevis
D.rerio
S.cerevisiae
S.pombe
C.fumosorosea
T.terrestris
M.thermophila
C.thermophilum
B
Maximum-Likelihood Phylogenetic Tree of CENP-H
99
52
59
81
38
90
96
H.sapiens
M.musculus
G.gallus
X.laevis
D.rerio
S.cerevisiae
S.pombe
C.fumosorosea
T.terrestris
M.thermophila
C
Maximum-Likelihood Phylogenetic Tree of CENP-K
99
54
76
97
47
83
82
99
H.sapiens
M.musculus
X.laevis
G.gallus
D.rerio
S.pombe
S.cerevisiae
T.terrestris
M.thermophila
C.thermophilum
C.fumosorosea
D
Absorbance at 280nm (mAU)
400
350
300
250
200
150
100
50
0
669
440
67
35
13.7
6
0
5
10
15
20
25
30
Elution volume (ml)
Loaded on SDS-PAGE
thCENP-K
thCENP-H
thCENP-I^NT
50kD
37kD
25kD

Figure S1

CENP-I Alignment

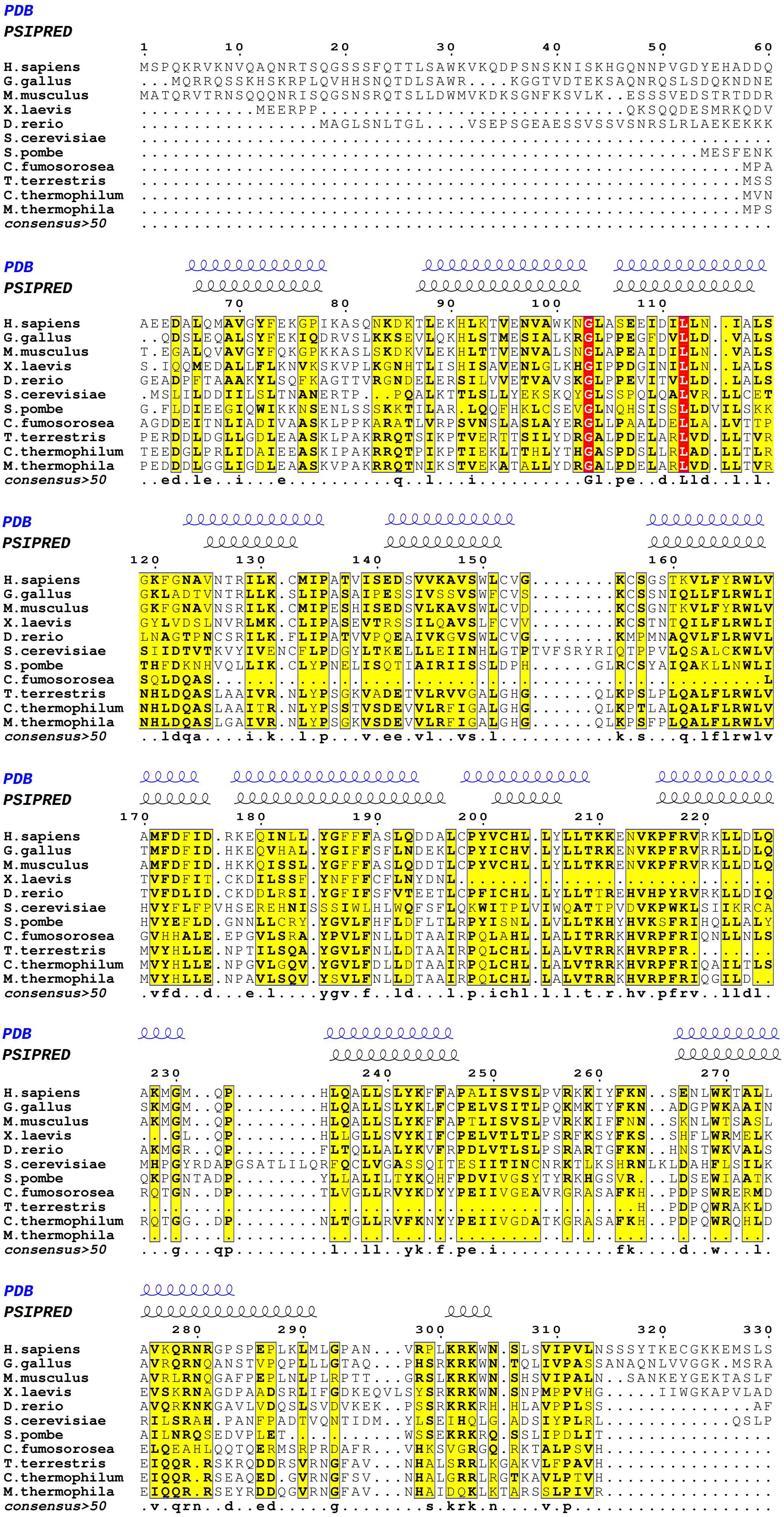

```
PSIPRED
                      340       350       360           370       380
H.sapiens        DCLNRSGSFPLEQLQSFPQLLQNIHCLELPSQ....MGSVLNNSLLLHYINCVRDEPVLL
G.gallus         DSYSANESFPVEQLRTFPQLLQNIHRLEFPSQ....MGSVLTNPLLLHYMNCSKDESVYL
M.musculus       DYLSSERSLPLEQLQRFPQLLESIHCLELPSQ....MCSVLNSPLLLHYINCVKDESILL
X.laevis         NPVSSEQMFPIERLQTFPQLLDNIHRLELPAQ....MGSVLKSPLLLHYVNCFKDDTPFL
D.rerio          DSNSSKKGLRLQELRSFKELLENIHNIELPAQ....MGSMLGSTLALHYLDCVQDESAFL
S.cerevisiae     EYVPSDSTVSLWDVTSLEQLAQNWPQLHIPNDVDYMMKPSLNSNVLLPRKVMSRDSLKHL
S.pombe          .MKNTSSSYSLEELTSVQQMGLVYEKIVFPSR....IAAVLKSKLFLIFLFLKNKNVYYS
C.fumosorosea    TTHATEDSVTLEEIENVQSFVDKMDKLELPNQ....LVAVLADPLLQKLLLLRPSAESYM
T.terrestris     TLHAQESSVTLEEIDSAESLAQNLEKVELPTQ....LVAVLADPLLQKLLLLRPDAEASS
C.thermophilum   TFHAQENSITLEEIDNAEAFVKNLEKFELPTQ....LVAVLADPLLQKLLLLRPSAEASS
M.thermophila    TLHAQESSVTLEEIDSAEAFARNLEKIELPSQ....LVAVLADPLLQKFLFLRPNAEASS
consensus>50     d....e.s..leelqs.eql.qn...lelP.q....m..vL.dplll.yl.l..de...l
```

```
PSIPRED
                  390         400       410       420       430
H.sapiens        RFYYWLSQTLQEE.......CIWYKV.NNYEHGKEFTNFLDTIIRAECFLQEGFYSCEAF
G.gallus         RLYYWMGQTLQEE.......CTWCVV.DNNQYEEFRGFLETVYKAECFLQEGFPSCEEF
M.musculus       RISYWLSQALQEE.......CVWYNI.NNYEQEKEFINFLDLVIRVQCFLQEGFYSCEAF
X.laevis         RLNYWLAFTLHEE.......CAWYK..GNKHSKEEVEAFLETVANTQQFLQEGLYSTEGF
D.rerio          RLNFWLGHSLQEE.......FLLCPVDGSSDNTSEACRFLNTILFSQQFLQEGFSSTESF
S.cerevisiae     YSSIILIKNSRDESSSPYEWCIWQLKRCFAHQIETPQEVIPIIISVSSMDNKLSSRIIQT
S.pombe          RLDEWLHITLNYG..........LALRSGSNNQEE..EVLHLLYKYLLFSPKFPKSLLQY
C.fumosorosea    RVANWLNAALQEV.......V......DGDADETTLWEMMDVVRDFVVQIKNLPATVLNF
T.terrestris     RISNWLVACLGDV.......A......SGDADVDLLLDMIEVIHDYAVATKTLPPLLLTF
C.thermophilum   RISSWLMACLGDV.......V......SGDADIELLLDMVEVIRDYVIATKGFPPLLLTF
M.thermophila    RISNWLMACLGDV.......A......SGDADSEIFLDMVEVIHDYVVATKTLPPILLTF
consensus>50     r...w$...lqee..................d.ee..e.le.i.....f.qe...s...f
```

```
PSIPRED
                440       450       460           470       480
H.sapiens        LY..KSLPLWDGLCCRSQFLQLVS.WIPFSS....FSEVKPLLFDHLAQLFFTST...IY
G.gallus         LY..RSLPLWDGVSCRSQILQLVS.WIPLST....FSEMKSQLCDPLAQLFFTSS...LY
M.musculus       LY..KSLPLWDGSSCRSQYLQLLA.WIPFSS....FSEVKPLLSDHLAPLFFTSS...IY
X.laevis         LY..KSLPHWNG.CHRSQILDLIS.WIPLGP....SSEMEELLYKPIAQIFISSS...VY
D.rerio          LY..KFLSVWDGSLLRPQILDLLS.DIPLMP....SFYMNKLLFEPLKQLYFTST...VF
S.cerevisiae     FCNLKYLKLDELTLKKVCGGILPL.WKPEL.....ISGTREFFVKFMASIFMWSTRDGHD
S.pombe          VI..TFFSKPNITEENYNLLTLLVTHIPITT....DSSYFNSLLKEFEQFILQKN...AE
C.fumosorosea    FA..HFFQLWNGEGSKTCIFQILV.YTPLHD....FDELYKHIFQPFEAAVADNK...PA
T.terrestris     LE..QFLRVWDGSGKRDMVLELLS.YVPLIDGANKQTELYKMILGPLEAAVLDNT...PS
C.thermophilum   FE..QFLHIWDD.............LSPLLN................LLETTLLDNT...PS
M.thermophila    FA..QFLRIWNGSEKRDIVFECLS.FAPLAE....FEELYKTL.QGLEHAVLDNT...PN
consensus>50     l....fl..w#g...r.qilqlls..iPl......sem...l.d.l...f..st.....
```

```
PSIPRED
                490       500       510            520       530       540
H.sapiens        FKCSVLQSLKELLQNWLLWLSM.....DIHMKPVTN.SPLETTLGGSMNSVSKLIHYVGW
G.gallus         FKCSVLESLKELLQNWLNWHVV.....QLDSESDSQFSSLNTTLSGLVNGVAELINFVGR
M.musculus       FKCSVLQSLQELLQNWLLWLST.....DAHVQPTTD.SPLETTLGGSMSSVSQLIEYTGW
X.laevis         FKCSILESLKALLQNWLIWHSF.....SVERMQTQS.INMNSTMSGLLTSVKDLIHFIGH
D.rerio          FKCSVIECLNSMLVKWLTWHSVSASEGSLDISLNSN.STMTMTLSGFMDSVMELVRFVGL
S.cerevisiae     NNCTFSETCFYVLQMITNWVL.............DDKLIALGLTLLHDMQSLLTLDKIFNN
S.pombe          FCSKHLNILWL.....WLFRML......NLRIASMGNNHTLLEKCLLITNHATFLVSHFSW
C.fumosorosea    TQLELLNMYTNLVHHWTSVL.................RSSKNIPAHASRTITSTVQHAGT
T.terrestris     SQVALLKLYTLLLRRWTVAM.................EAASDVAALAVASIPSLIDHVNK
C.thermophilum   SQLSLLTFYHRLLLHWTTLLL................SSPDPGQYPAGTSIPQLISHVNK
M.thermophila    SQVSLLKFYTSLLRRWTIVM.................EAANGVDTLPWATIPRLIDYVNQ
consensus>50     f.cs.le.l..ll..wl.wl...................ln.tl.....sv..li.yvg.
```

```
PSIPRED
                      550       560       570          580       590
H.sapiens        LSTTAMRLESNNTFLLHFILDFYEKVCDIYINYNLPL...VVLFPPGIFYSALLSLDTSI
G.gallus         ISTAALHLEKSHTFLLYFILDFYETVCDIYLKYKLPL...LIMPPAGVFYPALLSMDSVN
M.musculus       LCVVAMRLESSSTLLLHFILDFYEKVCDIYINYDLPI...VVLFPPVIFHSALLSLDATI
X.laevis         LSTLGLQI.HNSSLLLHFVLDIYELVSDMYVRFSLPL...VVLPPPGVFYPALLSTDCVN
D.rerio          LASEGLRLENCHVLLLNQTLAFYETVCDMFLKYSLPL...IIMPPAGVFYPALFAIDSIS
S.cerevisiae     ATSNRFST.MAFISSLDILTQLSKQTKSDYAIQYLIV........GPDIMNKVFSSDDPLL
S.pombe          DVSLAYQL.......SRLFQLYYKILTKIRKQIEPN.....IPPKELIYVLFFQPSAFY
C.fumosorosea    LALALLQT.GPTLSSESAILALYEQNLALLTDEALRSRVRIQLPPSALIYLLVLSQSLAT
T.terrestris     LSLTLTQT.SRTVGTYLDILDFYTAVTALYSKPALLQHVEIAIPPPLLVYLLFFCPSLAV
C.thermophilum   LALTLSQT.SPTVHTHLSILDFYASCANLYSNPALLSHISITIPHPLLVYNLYFSPSLAV
M.thermophila    LALTLTQT.SPTVGTQLVILDFYGASAVLYSSKKLLQHIEITIPPPLLVYLVYFSPSLAV
consensus>50     l...lq.......l..ildfye.v.dly...lp...i.lppp.i.y...s.d...
```

```
PSIPRED
                600       610             620       630       640       650
H.sapiens        LNQLCFIMHRYRKNLTAAKK.......NELVQKTKSEFNFSSKTYQEF.NHYLTSMVGCL
G.gallus         LNQLCYIMYRYRTNLIAAKE.......NEMSKKKIQQFKFSSQTYQEY.NQYIIAMVGCL
M.musculus       LNQLCYIMYRYRNNWTAAKK.......NRYLQKAKPEFSLSSKICKEY.NYYLTAMVCCL
X.laevis         LNQLCYIMYRYRTNLVTAKT.......NERQIKAQMHLNIHSHTFQEF.NKYLTAMVGCL
D.rerio          LDRLGYIMYRYRMNLTLAKQ.........QKQQREQTFHISRNAYKEF.NRNLQTMVNSM
S.cerevisiae     LSAACRYLVATKNKLMQYPS.....................TNKFVRMQNQYIMDLTNYL
S.pombe          INSMVGLLLLTKNYQERLMD...................SRIDAISKFTHSYLKSLSEII
C.fumosorosea    VARLCRIMASYKRGFETAMK......VRAGAGTTVDASSYSQDEVNRY.NGNLLDIVNLL
T.terrestris     ASRLCGILARYKRAWEAVMS.........SAVARKLTRPERDRIQAF.NGFLMDISNCV
C.thermophilum   VSRLCGILAMYKRAWEAVMASPSSSSSGSGSTVKRQLTKHERAQVNDF.NGFLMDLCNCL
M.thermophila    ASRLCGILASYKRAWETVMS.........PAVARQLTRRERDQVNVF.NGFLMDLCNCL
consensus>50     ln.lc.i$.ryk....a......................d..qef.n.yl..mvncl
```

```
PSIPRED
                              660         670                  680       690
H.sapiens       WTSKPFGK.......GIYID..PEILEKT.........GVAEYKNS..LNVVHHPSFLSY
G.gallus        WTSSAFQKDNHP..EGIRLD..DELLKKT.........GVREYKNS..FNIVYHPALMCY
M.musculus      WTSRPFKA.......GVYTD..PETIENT.........GGTQYKST..LNIVYHPSLLSY
X.laevis        WTSHAFHQDTHP..QGIQLE..THLLESS.........GVLTYKKA..FNIVYHPALIGY
D.rerio         WNSQWCIP.......GSDLEINKDLIALS.........KIAEPWCC..FDIIHHPALFSY
S.cerevisiae    YRNKVLSSKSLF...GVSPDFFKQILENLYI.......PTADFKNAKFFTITGIPALSYI
S.pombe         LLKEKRAILSFLQLWEPFKSDYSQFLPIATRIA.....NDHPYAQR.VFSLTCAPQFFSY
C.fumosorosea   WRMHAFGVERGDVEQGCMLP..AAARERLGRYAAAADRGVVTLAAA..LSLSYSPVLCLQ
T.terrestris    WRGRAFSTTDENA.QGCCVP..QSIQPRLESYLRAAD.SDLSLATA..FNLSHSPLLCLQ
C.thermophilum  WRGRAFTTTDLNA.RGCLLP..QSVQTVLEKYVRAVD.SEMSLAVV..FGLSYAPTLCLQ
M.thermophila   WRGRAFSTTDVNA.QGCRIP..QPVQATLEAYMRTAD.PDLSLATA..FGLSHSPLLCLQ
consensus>50    w...af.........g...d...e..................y.....fni...P.l..y

PSIPRED
                       700              710
H.sapiens       AVSFLLQES.......PEERTVNVSSIR.GK................KW.......SWY.
G.gallus        AVDFLQQAW.......PDDTTFNFNLIK.GK................KW.......NWY.
M.musculus      AASFLLQES.......PEEMTEHLSSIQ.GK................KW.......NWY.
X.laevis        CVHFYHQIF.......SEDKQFRLQLIK.GR................LW.......NKY.
D.rerio         AIEFHQRCW.......PERKDVDLGSIKTGK................YW.......NWY.
S.cerevisiae    CIIILRRLETAE....NTKIKFTSGIIN.EETFNNFFRVHHDEIGQHGWIKGVNNIHDLR
S.pombe         INGYQIYLQQTN....PATGSIPLKPIQ.EETFGAFQSNLHLSD...SW.......EDFQ
C.fumosorosea   SSEALRALEVRRIAADEAVETRHAGPAS.QESLRRLATSGGIRL...GF.......NGYR
T.terrestris    SISLVRELEDLEA...DEIRARHGGPVT.QASLNQLANRGGLSL...SW.......QEYR
C.thermophilum  AISFMRELEDED....ESVQARHAGPVT.QASLARLARRGGLEL...SW.......QDYR
M.thermophila   SLSYIRQLEEDEM...EDLRARHAGPVT.QVSLGRLANRGGLAL...SW.......QEYR
consensus>50    .i.fl.qle........e......g.i..q..................w.......n.y.

PSIPRED
                  720         730       740       750
H.sapiens       ...LDYLFSQG..LQGLKLFIRSSVHHSSIPRAEGINCNNQY...
G.gallus        ...LRYLYGQG..LEGLKLFIESSINRVSKASQSKAEDEDEKV..
M.musculus      ...LDYLYSEG..FQGLKLFIKSSVHSSVPKPEENTE........
X.laevis        ...LEFLYSQG..LNGLKHFIESSVNRLASNSQQQEPCDLLSSKS
D.rerio         ...MEFLFNQG..FDGLNNFIRTNTGHGPSSSSQDDRQPLSQNCH
S.cerevisiae    VKILMHLSNTANPYRDIAAFLFTYLKSLSKYSVQNS.........
S.pombe         KNFIIYLKKKG..YLAISDFLLSTLNR..................
C.fumosorosea   ASVLEALRAKG..MPGVEELLKASMPSAAKAIESWAAGKEA....
T.terrestris    AAVLAHLESKG..LPGIPELMYNTMKNLMKARK............
C.thermophilum  VGVLEYLEAKG..FGGIKELMYNTMKNLMKERRT...........
M.thermophila   SGVLEYLEAKG..FGGISDLMYNTMKNLMRARRS...........
consensus>50    ...leyL...g..f.gi.efl...m....................
```

Figure S2

CENP-K Alignment

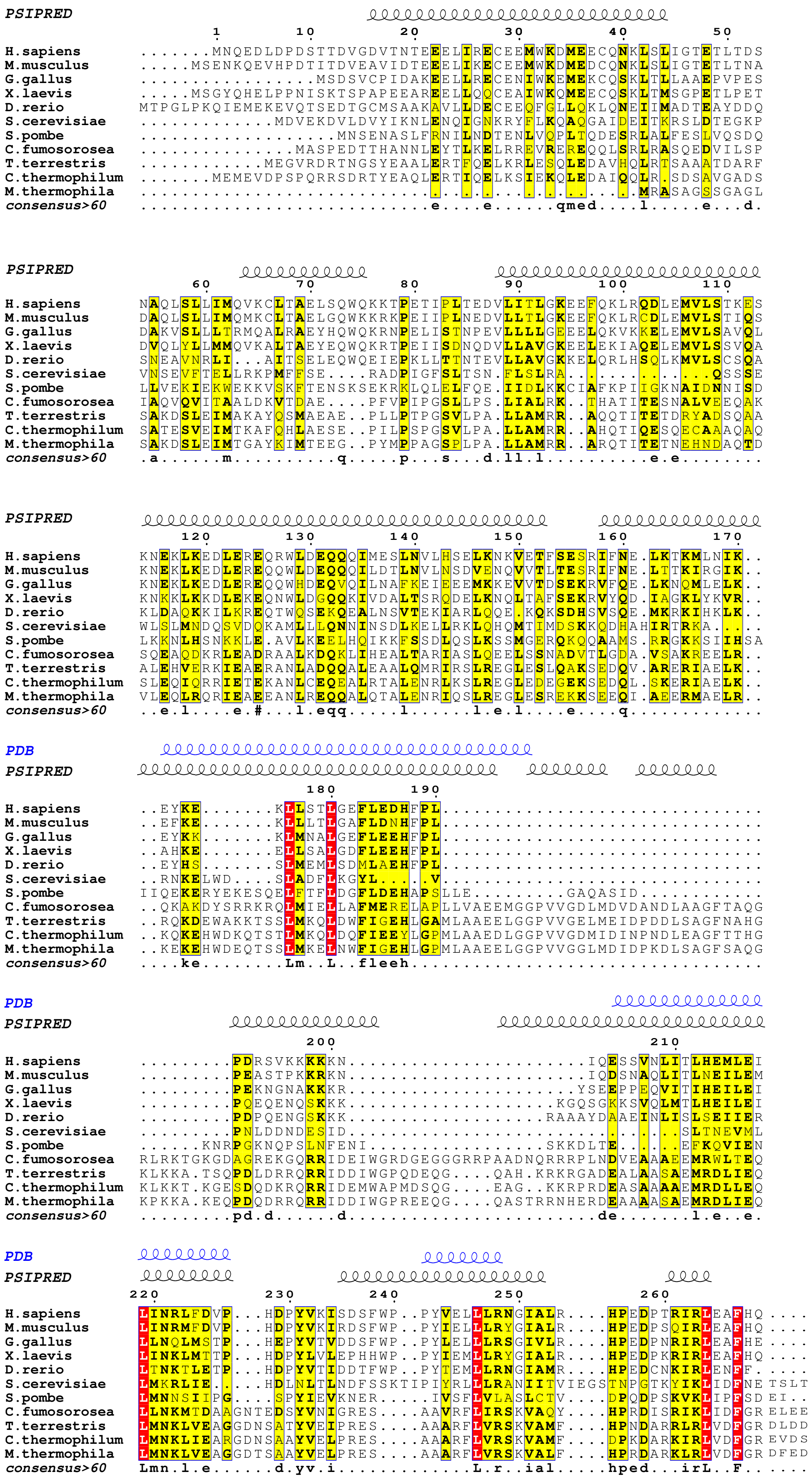

Figure S3

CENP-H Alignment

```
PSIPRED
                  1        10        20        30        40        50        60
H.sapiens         MEEQPQMQDADEPADSGGEGRAGGPPQVAGAQAACSEDRMTLLLRLRAQTKQQLLEYKSM
M.musculus        MEEQP..RERSEAGAEACEEKRG....LSQAAEERIEDRISLLLRLRAQTKQQLLEYKSM
G.gallus          ..MAGRLSESVGSGPGAE.........AETAADPDAKRDVLEHLCARTHLKQLVMEFDTA
X.laevis          MDLPGPLGDMQALSSELQRVCLLGACGQEPPEEEPPEPHLLRLLRLREQVKHQSLEVQTV
D.rerio           MSSSNVNHDALASDVDTPRRDNS....LPDPSSVSDGVTPTSLLKMKEIMENQCFEMNVK
S.cerevisiae      ...........................MTNSSEKQWERIQQLEKEHVEVYRELLITLDRL
S.pombe           .........MDNNDQISN.........LCLGALQLLADQPISVISNEPSEEQRNEHVLKK
C.fumosorosea     .......MDNDEHMEDVE.........SAGEPPLGLSDDERQVLELYDRLQQMDLEIA.I
T.terrestris      ..........MAATAST..........ATPTPLPHFTEREASVLALYDRLRQLQLELA.L
M.thermophila     ..........MASHSPS..........VSS...PAFTETEASVLALYDQLQQLQLELA.L
consensus>60      ........d..................d.....l.l.e...q..le....

PSIPRED
                          70        80        90       100       110       120
H.sapiens         VDASEEKTPEQIMQEKQIEAKIEDLENEIEEVKVAFEIKKLALDRMRLSTALKKNLEKIS
M.musculus        IDTNEEKTPEQIMQEKQIEVKIEELENEIEDVKSNIEMKSLALSRMKLSVALRDNMENMG
G.gallus          CPPDEGCNSG...AEVNFIESAKETLEEVGKVKSAFESKALVIKRIQLMDALRKRVKEND
X.laevis          AEASEEVSSEALSSEKLTEIAKELLK.DIEDLKVSYQNKTMVLQRMQFVNALLNKIQDNN
D.rerio           VSMGKHRES.....CEAEADLSKYESEIEQARLSHFNKTLVLNRMQIWSAVIEKMIQNG
S.cerevisiae      YLIRKHNHA..........VILSHTQQRLLEIR...HQLQINLEKTALLIRLLEKPDNTN
S.pombe           WENNVLRHW......KILFSLFMQTCGDSVQGNAAFSILN.FMTTNSLVGQLYQRLYDES
C.fumosorosea     INAQKAHVS...TSTDDSPDALAAAQEALLQARSRYKLRNDAVELVMMVHPVLKAVHGST
T.terrestris      LTSQRSHGVIETSASRRAENDLAVDEARLLEARATLDLRDSVVENVVTLQPILNAVHHAT
M.thermophila     LQSQHNVG......SPTTGHDLSENQTRFLEAKATLALCNSVVESVVAVQPTLNAVHQAR
consensus>60      .d.q...................e.e..e..........le...l...l.n...n..

PSIPRED
                          130       140        150       160        170
H.sapiens         RQSSVLMDNMKHLLELNKLIMKSQ...QESWDLEEKLLDIRKKRLQLKQAS...ESKLLE
M.musculus        PENCVLTDDMKHILKLQKLIMKSQ...EESSELEKKLLDVRKKRLQLKQAS...RSKLLE
G.gallus          GCARLIVETMRDIIKLNWEIIQAH...QQARVIRENLNDIRRKRYFLKQAE...GEKALR
X.laevis          NESRLIQEKFTHISTLSSAVINSQ...QETRELEDRLYDIRKKRLVLKELG...SAKMLE
D.rerio           ADAEELKELTNQNSEICEKTIQIL...KETRELQDQITDVQKQRLDLKGQI...KKKMQE
S.cerevisiae      VLFTKLQNLLEESNSLDYELLQSLG...AQSSLHKQLIESRAERDEL........MSKLIE
S.pombe           NSLGFISSPLNETEHLSEENLKIESSITFTSEEIEKEKENIKSVLLPSVQS...ISSSLE
C.fumosorosea     NASPIDRDLLPYIVKRDDAAVEVARAAAQAQATRDALTDVQVETLRACRRNVTLTSRLFE
T.terrestris      HASPLERDLLPYTEQRDATATKVASLCADLDSAWGRLAELETESLRATQRNMELASEVLR
M.thermophila     QASAVERDLLPIIQERDIAATKAANIYSELQTARARLAELEVGSLKASHWNRELAADVLQ
consensus>60      .......d.l.....ld...........e.....e.l.#i...rl.l........sklle

PDB
PSIPRED
                      180       190       200       210       220       230
H.sapiens         IQTEKNKQ.KIDLDSMENSERIKIIRQNLQMEIKITTVIQHVFQNLILGSKVNWAEDPAL
M.musculus        IQIEKNKQ.KEDVDKMENSEMIKTMKKKLQTEIKITTVVQHTFQGLILASKTNWAEDPAL
G.gallus          IFTTVRKK..KEVVRMKIAEKLKFIHRNVQYERKVTTLVQNILQNIIVGCQINWAKDPSL
X.laevis          IQTLKSNW.KANREETQN.EKIKRLNKILKDEIDSTTVLQNVFQNIILASHVDWAKDPQL
D.rerio           INELKQEK...ENQGEVQQKTKERAEAVLQKYQRITTILQNVLRGIILASKVNWREDPKL
S.cerevisiae      LSSKFPKP............TIPPDDSDTAGKQVEVEKENETIQELMIALQIHSGYTNIS
S.pombe           LYASKESD.........YRSSIEGVLADLKLARARWEIVRNVTQILLLESGISFLENKKL
C.fumosorosea     LAGQLEERRAVDWHGG.......EEALRVREEKRKWRAVKGAASGIVAGSGVDWASDEAL
T.terrestris      LAAGTNDKTPSQLEGGRLEMELAGHEAEMKSNRRRWRIMKGAASAIVAGSGIDWVRDERL
M.thermophila     LASENHKQDFESIQNGRFGSGIADLESQVKSSRHRWRIIKGATSAIIAGSGIDWVRVKRL
consensus>60      .....n.....d.d......i.....nl..e.....ivq...q.ii..s.inw..d..l

PDB
PSIPRED
                        240
H.sapiens         KEIVLQLEKNVDMM...
M.musculus        RETVLQLEKDLNTL...
G.gallus          RAIILQLEKDISIQNLL
X.laevis          TDIVLKLETNASSYT..
D.rerio           SDIVMKLEHIPS.....
S.cerevisiae      YTI..............
S.pombe           AYIMDLCGDREDNYSSY
C.fumosorosea     REMVLDPEDDD......
T.terrestris      RDLVLDLPD........
M.thermophila     RELVLDPLD........
consensus>60      .eivlqle.........
```

Figure S4

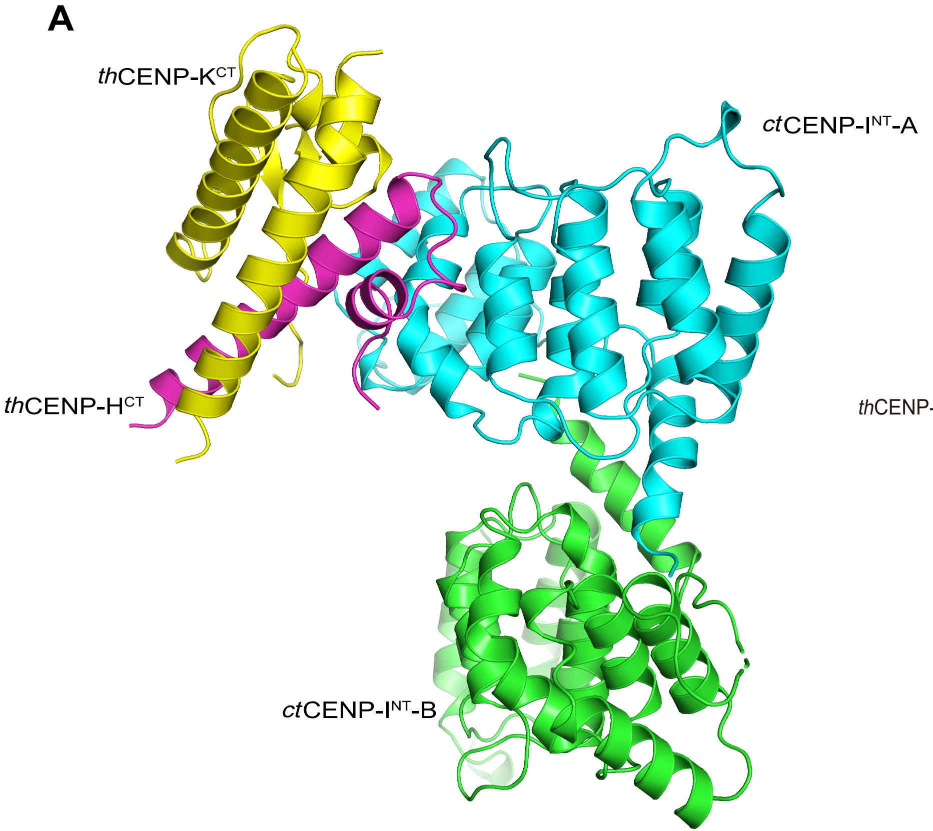

A
*th*CENP-K^CT
*ct*CENP-I^NT-A
*th*CENP-H^CT
*ct*CENP-I^NT-B

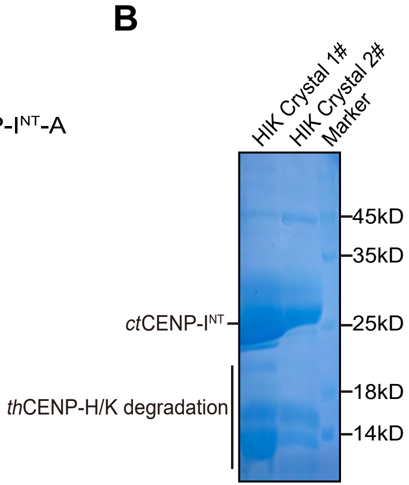

B
HIK Crystal 1#
HIK Crystal 2#
Marker
45kD
35kD
25kD
18kD
14kD
*ct*CENP-I^NT
*th*CENP-H/K degradation

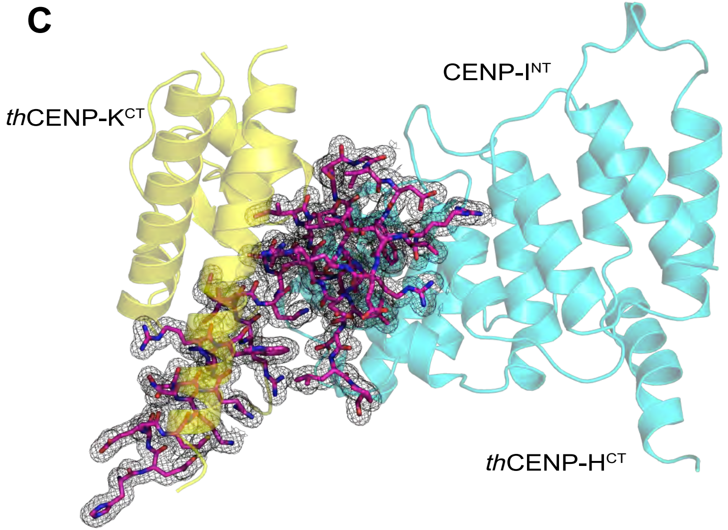

C
CENP-I^NT
*th*CENP-K^CT
*th*CENP-H^CT

Figure S5

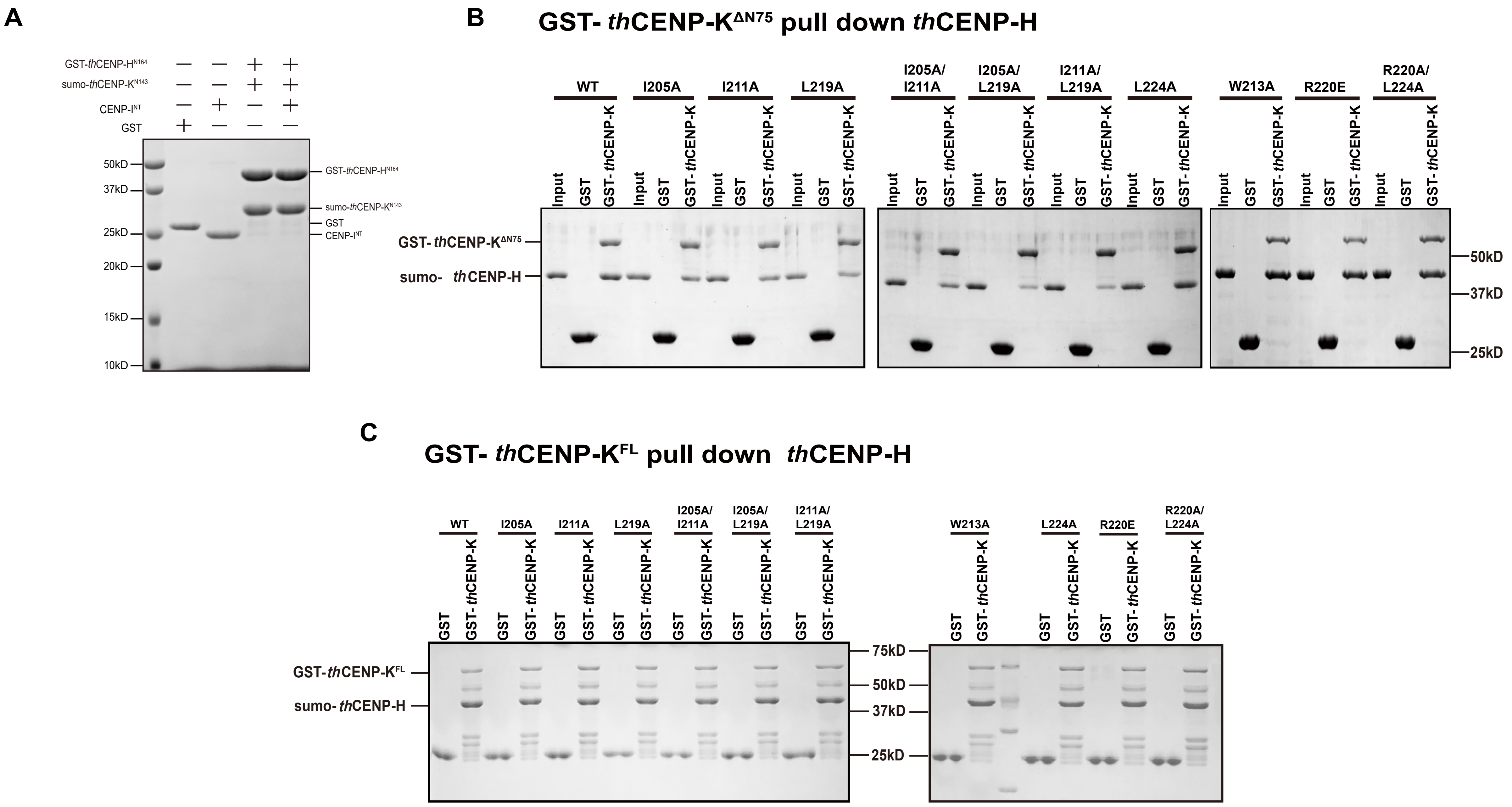
A
GST-thCENP-H^N164
sumo-thCENP-K^N143
CENP-I^NT
GST
50kD
37kD
25kD
20kD
15kD
10kD
GST-thCENP-H^N164
sumo-thCENP-K^N143
GST
CENP-I^NT
B
GST- thCENP-K^ΔN75 pull down thCENP-H
WT
I205A
I211A
L219A
I205A/ I211A
I205A/ L219A
I211A/ L219A
L224A
W213A
R220E
R220A/ L224A
Input
GST
GST- thCENP-K
GST- thCENP-K^ΔN75
sumo- thCENP-H
50kD
37kD
25kD
C
GST- thCENP-K^FL pull down thCENP-H
GST-thCENP-K^FL
sumo- thCENP-H
75kD
50kD
37kD
25kD

Figure S6

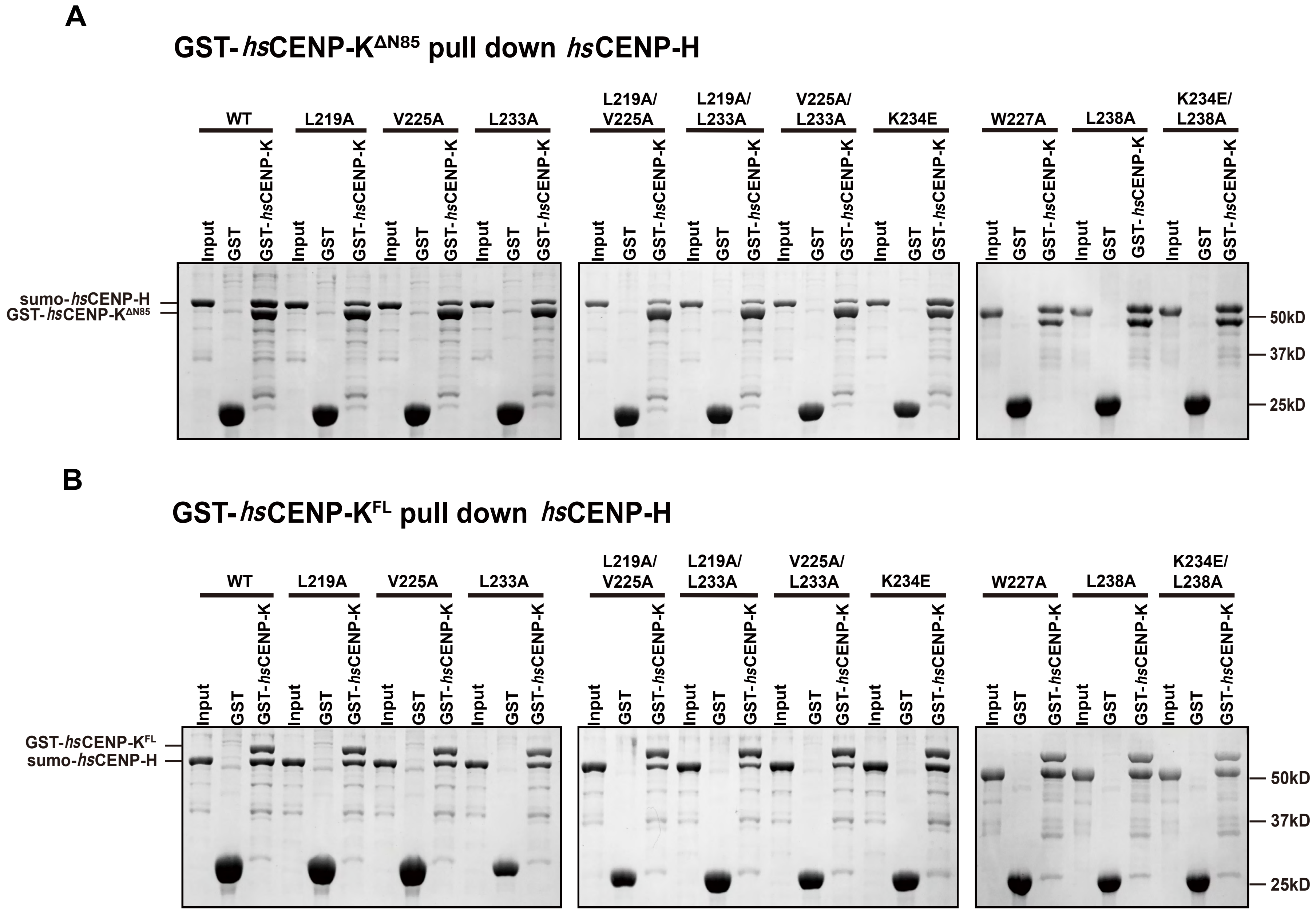
A
GST-hsCENP-K^ΔN85 pull down hsCENP-H
WT
L219A
V225A
L233A
L219A/V225A
L219A/L233A
V225A/L233A
K234E
W227A
L238A
K234E/L238A
Input
GST
GST-hsCENP-K
sumo-hsCENP-H
GST-hsCENP-K^ΔN85
50kD
37kD
25kD
B
GST-hsCENP-K^FL pull down hsCENP-H
GST-hsCENP-K^FL
sumo-hsCENP-H

Figure S7

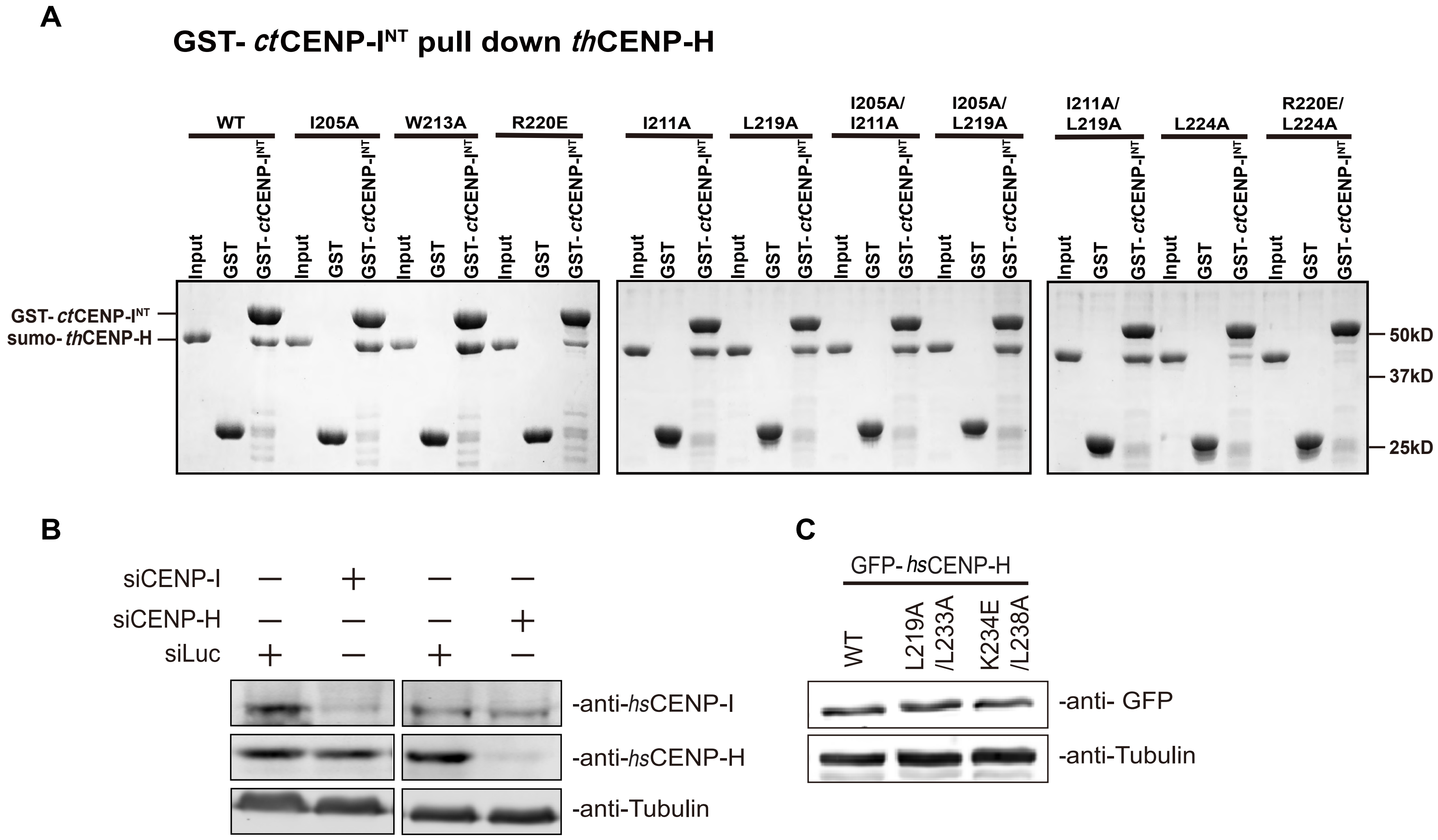
A
GST- ctCENP-I^NT pull down thCENP-H
WT
I205A
W213A
R220E
I211A
L219A
I205A/ I211A
I205A/ L219A
I211A/ L219A
L224A
R220E/ L224A
Input
GST
GST- ctCENP-I^NT
GST- ctCENP-I^NT
sumo- thCENP-H
50kD
37kD
25kD
B
siCENP-I
siCENP-H
siLuc
-anti-hsCENP-I
-anti-hsCENP-H
-anti-Tubulin
C
GFP- hsCENP-H
WT
L219A /L233A
K234E /L238A
-anti- GFP
-anti-Tubulin

Figure S8
